# Characteristics of Circular RNA Expression Profiles of Porcine Granulosa Cells in Healthy and Atretic Antral Follicles

**DOI:** 10.3390/ijms21155217

**Published:** 2020-07-23

**Authors:** Li Meng, Katja Teerds, Jian Tao, Hengxi Wei, Marcel Jaklofsky, Zhihong Zhao, Yaodi Liang, Li Li, Chi Chiu Wang, Shouquan Zhang

**Affiliations:** 1 National Engineering Research Center of Breeding Swine Industry, College of Animal Science, South China Agricultural University, Guangzhou 510642, China; taojian19922020@gmail.com (J.T.); weihengxi@scau.edu.cn (H.W.); 20190772@m.scnu.edu.cn (Z.Z.); liangyaodi@stu.scau.edu.cn (Y.L.); lili007@scau.edu.cn (L.L.); 2 Guangdong Provincial Key Lab of Agro-Animal Genomics and Molecular Breeding, and Key Lab of Chicken Genetics, Breeding and Reproduction, Ministry of Agriculture, South China Agricultural University, Guangzhou 510642, China; 3 Department of Obstetrics & Gynaecology; Li Ka Shing Institute of Health Sciences; School of Biomedical Sciences, The Chinese University of Hong Kong, Shatin, Hong Kong; ccwang@cuhk.edu.hk; 4 Human and Animal Physiology, Wageningen University, De Elst 1, 6708 WD Wageningen, The Netherlands; katja.teerds@wur.nl (K.T.); marcel.jaklofsky@wur.nl (M.J.)

**Keywords:** circRNA expression profiles, antral follicular atresia, granulosa cell apoptosis

## Abstract

Circular RNAs (circRNAs) are thought to play essential roles in multiple biological processes, including apoptosis, an important process in antral follicle atresia. We aimed to investigate the potential involvement of circRNAs in granulosa cell apoptosis and thus antral follicle atresia. CircRNA expression profiles were generated from porcine granulosa cells isolated from healthy antral (HA) and atretic antral (AA) follicles. Over 9632 circRNAs were identified, of which 62 circRNAs were differentially expressed (DE-circRNAs). Back-splicing, RNase R resistance, and stability of DE-circRNAs were validated, and miRNA binding sites and related target genes were predicted. Two exonic circRNAs with low false discovery rate (FDR) high fold change, miRNA binding sites, and relevant biological functions—circ_CBFA2T2 and circ_KIF16B—were selected for further characterization. qRT-PCR and linear regression analysis confirmed expression and correlation of the targeted genes—the antioxidant gene *GCLC* (potential target of circ_CBFA2T2) and the apoptotic gene *TP53* (potential target of circ_KIF16B). Increased mRNA content of *TP53* in granulosa cells of AA follicles was further confirmed by strong immunostaining of both p53 and its downstream target pleckstrin homology like domain family a member 3 (PHLDA3) in AA follicles compared to negligible staining in granulosa cells of HA follicles. Therefore, we concluded that aberrantly expressed circRNAs presumably play a potential role in antral follicular atresia.

## 1. Introduction

Each estrous cycle, a cohort of small antral follicles in, is recruited to enter the pool of growing antral follicles. From this pool, dominant follicles are selected by a process highly dependent on follicle-stimulating hormone (FSH) [1,2]. Follicular development is not a very efficient process as, depending on the species, around 90–99.9% of ovarian follicles degenerate before reaching the ovulatory stage by a process named atresia. In the adult female, atresia ensures that only the healthiest follicles, containing oocytes of optimal quality, will ovulate [1,3]. Besides, follicular atresia is also a major contributing factor to ovarian aging, characterized by the quantitative and qualitative decrease in ovarian follicles [4].

Granulosa cell death by apoptosis is the main cause of antral follicular atresia [5]. Initiation of granulosa apoptosis can be triggered by extrinsic factors, such as inflammation, and intrinsic factors, such as oxidative stress [6]. Under the condition of oxidative stress, pro-apoptotic factors, like the transcription factor *TP53*, may become activated, inducing cellular apoptosis through activation of caspases [7]. Until now, the underlying regulatory mechanism, which determines whether granulosa cells of selected antral follicles will undergo apoptosis, is still not fully characterized.

Noncoding RNAs, such as miRNAs, have been emerged in the past 10 years as regulatory molecules involved in many physiological and pathological processes, including oxidative stress and apoptosis [8]. In the ovary, miRNAs are involved in apoptosis of granulosa cells via regulation of gene expression at the post-transcriptional level by either inducing mRNA degradation or suppression of protein translation [9]. miR-493-3p, for example, can induce apoptosis in ovarian cancer cells through down-regulation of several targeted genes and activation of caspases, resulting in DNA fragmentation [8]. A more recently discovered class of non-coding RNAs, so-called circular RNAs (circRNAs), is emerging as another class of important modulators of cellular and organ functioning. CircRNAs are characterized by a covalently closed-loop structure in which a downstream 5′ splice site (ss) is joined with an upstream 3’ ss, generated by precursor mRNA back-splicing of exons of thousands of genes in eukaryotes [10]. The resulting RNA circle is ligated by a 3’-5’phosphodiester bond at the junction site [11,12]. CircRNAs are resistant to RNase R and very stable with longer half-lives compared to linear forms [13]. CircRNAs were originally considered aberrant splicing byproducts with little functional potential [14]. More recently, studies have demonstrated that some circRNAs potentially play important roles in both normal physiological and pathological conditions, such as immune responses and cancer development [10]. The functions of most circRNAs remain largely unexplored. Known functions include modulation of transcription and interference with splicing, and, in some cases, translation to produce polypeptides. CircRNAs can act as a miRNA sponge to regulate the targeted gene expression, which is the most widely investigated function of circRNAs up to date [10].

Regarding their role in apoptosis, circRNAs have been shown to be involved in either preventing or stimulating cellular apoptosis in different tissues via sponging miRNAs. For example, circLMO7, identified in bovine muscle tissue, promotes proliferation of myoblasts and protects these cells from apoptosis, possibly by sponging of miR-378a-3p [15]. Similarly, in vitro experiments using hydrogen peroxide to stimulate apoptosis have shown that knockdown of circ-ABCB10 has significantly suppressed proliferation and increased apoptosis of breast cancer cells due to the absence of sponging of miR-1271, indicative of a role of circ-ABCB10 in inhibiting apoptosis [16]. In contrast, circNCX1, which is up-regulated in the presence of oxidative stress, promotes apoptosis of cardiomyocytes by acting as an endogenous miR-133a-3p sponge [17].

Recent studies have demonstrated that circRNAs may play important roles in ovarian follicle development. Fu et al. [18] showed that the expression levels of some circRNAs altered in bovine cumulus cells treated with bone morphogenic protein 15 (BMP15) and growth differentiation factor 9 (GDF9). Another study reported the presence of circRNAs in preovulatory follicles of goats [19], while Cheng and colleagues reported 56 differentially expressed circRNAs in human granulosa cells obtained from a relatively young (≤30 years) compared to a more advanced age group (≥38 years), suggesting a possible involvement of circRNA in ovarian aging [20]. In line with these results, RNA-seq data suggest that circRNAs may play a role in polycystic ovary syndrome (PCOS), and, consequently, its use as a PCOS biomarker has been proposed [21].

In the present study, we intended to address the question of whether there is a role for circRNAs in the process of granulosa cell apoptosis. Due to the easy accessibility to large quantities of porcine ovarian tissue, we chose to use the pig as an animal model to investigate the role of circRNAs in antral follicular atresia. For this purpose, the features of circRNAs in porcine granulosa cells derived from healthy and atretic antral follicles were studied using RNA-seq, followed by qRT-PCR in combination with circRNA-miRNA-mRNA network construction and validation.

## 2. Results

### 2.1. Histological and Hormone Characteristics between Healthy Antral (HA) and Atretic Antral (AA) Follicles

In antral follicles with a well-vascularized follicular wall and clear follicular fluid, the cells from the granulosa layer were tightly adhered to each other, indicative of a healthy antral follicle. In follicles with a poorly vascularized follicular wall and a rather opaque appearance, the granulosa cell layer was disorganized, and numerous apoptotic cells were present; in some cases, apoptotic granulosa cells were found to be dispersed throughout the antrum, indicative of follicular degeneration. No cleaved caspase 3 (cCASP3) immunostaining was observed in granulosa cells of healthy antral follicles (Figure S1A), while clear staining was observed in granulosa cells of atretic antral follicles (Figure S1B). On the contrary, no morphological differences in the oocyte surrounding cumulus cells were observed between HA and AA follicles.

**Figure 1:**
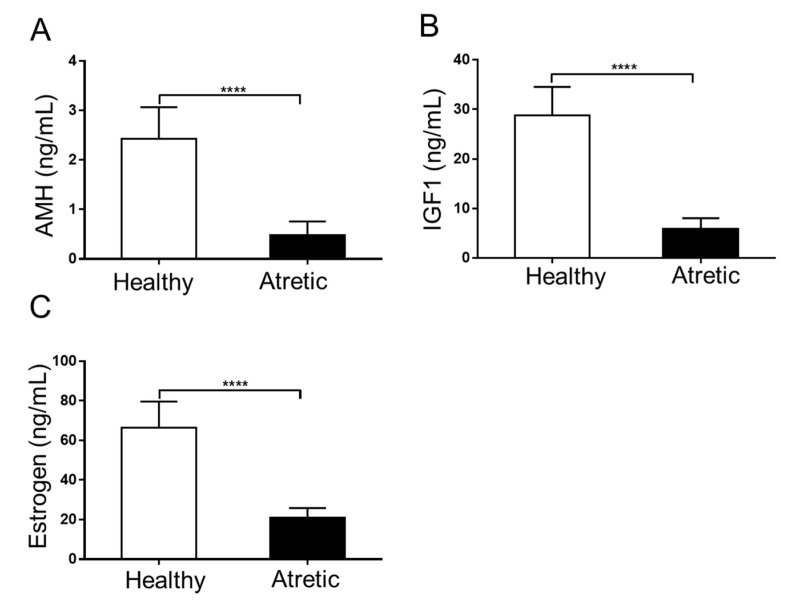
Hormone concentrations in pooled follicular fluid of 4–7 mm HA and AA follicles obtained from 6 different sows. (**A**) Anti-Müllerian hormone (AMH) concentrations (ng/mL). (**B**) Insulin-like growth factor 1 (IGF1) concentrations (ng/mL); (**C**) Estradiol concentrations (ng/mL). HA, healthy antral follicles; AA, atretic antral follicles. Values represent means +/− SEM. ****, *p* < 0.001.

Anti-Müllerian hormone (AMH) and Insulin-like growth factor 1 (IGF1) concentrations (Figure 1A,B) were both significantly decreased in follicular fluid of AA follicles compared to HA follicles. Concomitantly, estradiol concentrations were significantly lower in the follicular fluid of AA follicles (Figure 1C). These results further supported the correctness of the macroscopical classification employed to discriminate between HA (well-vascularized) and AA (opaque colored) follicles.

After scraping the cells from the follicular wall, as described in the Materials and Methods, 98.0 ± 1.4% of the cells could be identified as granulosa cells based on positive staining with the follicle stimulating hormone receptor (FSHR) antibody. Furthermore, to ensure that the isolated granulosa cells were not contaminated with theca cells, the mRNA content of cytochrome P450 family 17 subfamily A member 1 (CYP17A1), a specific theca cell marker, was measured by qRT-PCR (see below) [22]. CYP17A1 levels were below the detection limit of the assay, implicating that theca cell contamination was negligible, further confirming the purity of the granulosa cell isolates, which were next used for RNA-seq.

### 2.2. Profiles and Characteristics of circRNAs in Granulosa Cells of Ovarian Antral Follicles

**Figure 2:**
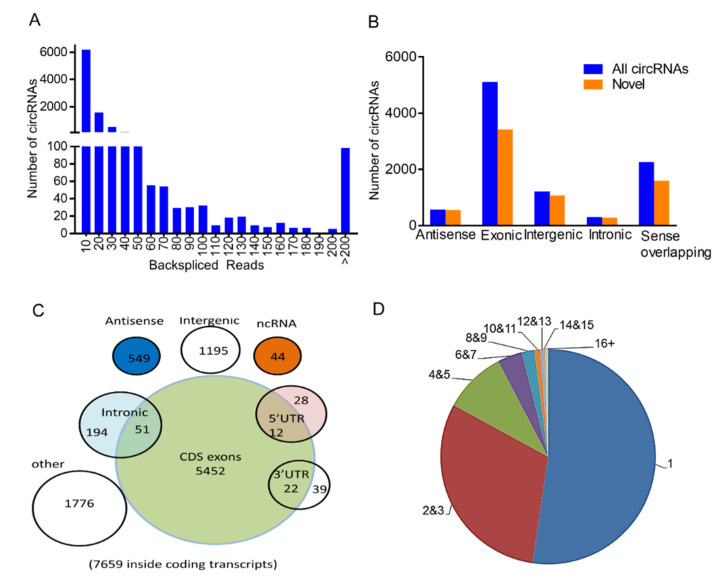
Expression profiles of circular RNAs (circRNAs) in porcine granulosa cells in HA and AA follicles. (**A**) A number of circRNAs with different back-spliced reads. (**B**) A number of newly discovered circRNAs among different types of circRNAs. (**C**) Genomic origin of identified porcine circRNAs. (**D**) A number of circRNAs produced from one gene. HA, healthy antral follicles; AA, atretic antral follicles. Follicles were isolated in batches at three different time points, each batch containing 15–20 ovaries.

In total, 9362 distinct circRNAs containing at least two unique back-spliced reads (Figure 2A) were identified based on the circRNAs identification standard [23]. Although 67.3% of circRNAs had average coverage of less than 10 unique back-spliced reads, there were 441 circRNAs highly expressed containing a high-average read count of more than 50, including circ_KIF16B, circ_IL1R1, circ_SLC30A7, and circ_ANGPT1. We identified 2515 overlapping circRNAs and 6847 novel circRNAs that had not been identified so far (Figure 2B; [24,25]). The 54.36% (5090/9362) of circRNAs identified were derived from exons and generated from individual linear cognate genes. The remainder were annotated to sense, among others, overlapping (2243/9362) and intergenic regions (1195/9362). All exonic circRNAs were spliced from protein-coding exons (Figure 2C). The length of most exonic circRNAs was less than 1300 nucleotides (nt), with a median length of ~900 nt (Figure S2A). CircRNAs were widely distributed across all chromosomes, with chromosome 1 containing the highest number of circular transcripts, namely, >1000 circRNAs, including 709 novel transcripts (Figure S2B).

Further analysis revealed that most host genes could only produce one single circRNA; nevertheless, there were a number of genes that produced multiple circRNAs (Figure 2D, 9362 circRNAs from 3515 host genes). Seventy-two host genes generated more than 10 different circRNAs per gene. The most striking example was the 36 exons containing HEAT repeat containing 5A (HEATR5A) located on chromosome 7, which generated 54 distinct circRNAs (with at least two unique back-spliced reads). The abundance of these 54 isoforms was further investigated, and it appeared that one dominant isoform was significantly higher expressed than the other 53 isoforms. Similarly, 36% of the host genes (26/72) with more than 10 distinct circular isoforms also showed significantly higher expression compared to the other isoforms. This suggested that in gene loci with more circRNA isoforms, one isoform seemed to predominate over the other circRNA isoforms linked to this gene.

### 2.3. Identification of Differentially Expressed circRNAs (DE-circRNAs)

**Figure 3:**
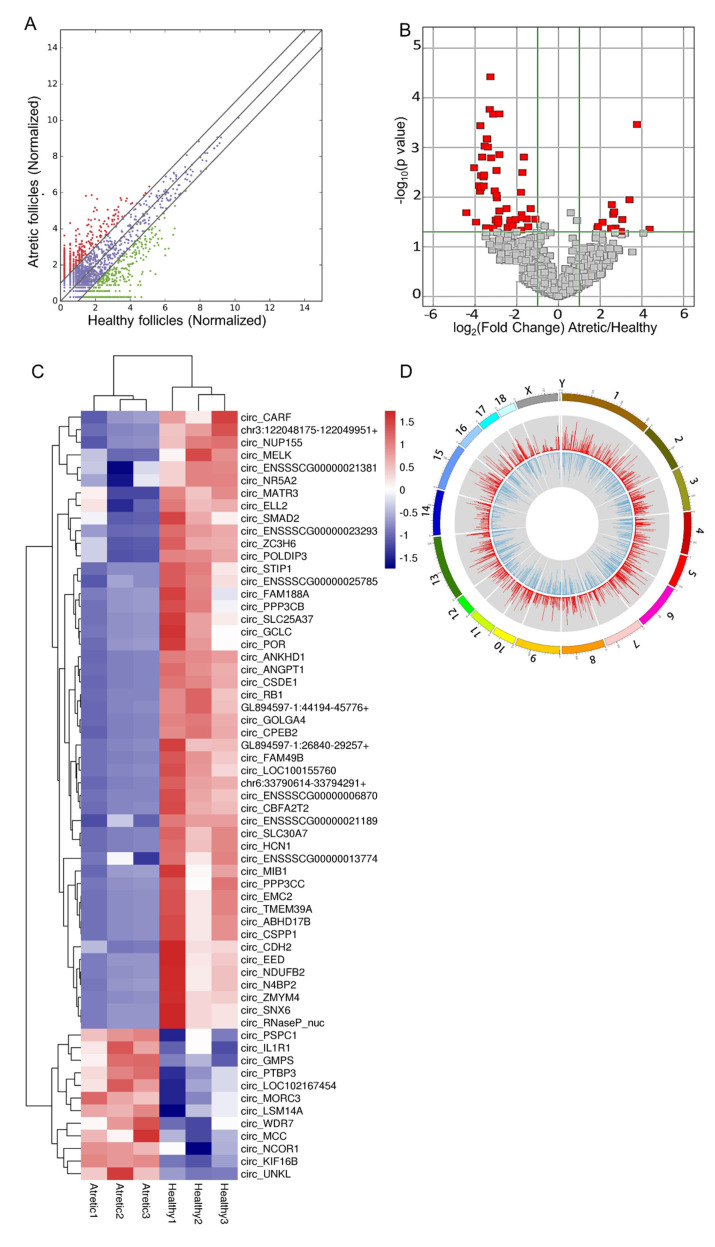
Profiles of differentially expressed circRNAs (DE-circRNAs). (**A**) Scatter plot, assessing the overall distribution of DE-circRNAs between HA and AA follicles. Values on the horizontal and vertical axis represent the averaged normalized signal values of each group (log_2_ transformed). The red and green dots indicate circRNAs with fold change (FC) ≥ 2.0. (**B**) Volcano plot, visualizing the statistical difference of the DE-circRNAs. The horizontal axis represents the FC of detected circRNAs, and the vertical axis represents the *p*-value. The red dots in the plot denote the significantly different DE-circRNAs (FC ≥ 2.0 and *p* ≤ 0.05, respectively). (**C**) Hierarchical clustering, showing the expression profiles of all the DE-circRNAs. Rows represent circRNAs, while columns represent different samples. The circRNAs were classified according to Pearson correlation. (**D**) Circos plot, showing the distribution of DE-circRNAs on porcine chromosomes. From the outside to the inside, the circos plot was divided into three layers. The first layer indicated the chromosome map of the porcine genome, where the DE-circRNAs were located. The second layer with red bars denoted the mean expression levels of DE-circRNAs in AA follicles, while the third layer represented the average expression levels of DE-circRNAs in HA follicles. CDS, protein-coding exons. Follicles were isolated in batches at three different time points, each batch containing 15–20 ovaries.

The analysis of circRNAs expression showed that there were 62 circRNAs significantly differently expressed between HA and AA follicles (Table 1, of which 49 circRNAs were significantly down-regulated, and 13 circRNAs were significantly up-regulated in AA follicles (fold change ≥ 2 and FDR ≤ 0.05, Figure 3A,B). All up-regulated and down-regulated circRNAs were further evaluated by unsupervised hierarchical clustering analysis (Figure 3C), which showed a clear distinction in samples of HA and AA groups. Most DE-circRNAs were spliced from exons; over 84.37% of exonic circRNAs consisted of 2–6 exons, while a smaller fraction of DE-circRNAs was spliced from sense overlapping, intergenic, and antisense regions of the genome (Figure S2C). The size distribution of the exonic DE-circRNAs was, in general, less than 1000 nt, while the length of the DE-circRNAs from sense overlapping regions was more than 3000 nt (Figure S2D). In addition, DE-circRNAs were widely distributed across chromosomes 1–18 and the X chromosome (Figure S2E). The distribution of DE-circRNAs was not uniform among different chromosomes; there was a general trend that numbers of DE-circRNAs per chromosome increased with absolute chromosome length (Figure 3D).

### 2.4. GO and KEGG Analysis of Host Genes of DE-circRNAs

For the biological process (Figure S3A, Table S1), five of the top 10 gene ontology (GO) terms were involved in gene expression-related processes, including gene expression, RNA metabolic process, transcription and DNA-templated synthesis, RNA biosynthetic process, and nucleic acid-templated transcription. Three others of 10 GO terms were related to homeostasis-related processes, including regulation of the homeostatic process, cellular homeostasis, and regulation of ion homeostasis. Regarding molecular function, the dominant categories were organic cyclic compound binding, heterocyclic compound binding, and metal ion binding, respectively (Figure S3B). For cellular components, the dominant processes were centrosome, transcription elongation factor complex, and protein serine/threonine phosphatase complex (Figure S3C). Kyoto encyclopedia of genes and genomes (KEGG) pathway analysis of the host genes of these DE-circRNAs showed that apoptosis was one of the significantly enriched pathways (Figure S3D, Table S2).

The underlying host genes related to GO processes and KEGG pathway analysis were similar. The enriched pathways included seven repetitive host genes of the DE-circRNAs (Table S2). Among these genes, three host genes appeared in most pathways, namely, *PPP3CB*, *RB1*, and *SMAD2*. In addition, *PPP3CB* enriched in the apoptosis-related pathway also appeared in nine out of the top 10 GO processes (Table S1), while *RB1* and *SMAD2* were present in six out of the top 10 GO processes. *IL1R1* was present in three pathway maps, including apoptosis, *CPEB2*, *NDUFB2,* while *NR5A2* appeared in only one pathway map. *CPEB2* and *NR5A2* were also present in the top 10 GO processes. These results indicated that the GO analysis was consistent with the pathway analysis.

### 2.5. Validation of DE-circRNAs Using qRT-PCR Combined with RNase R

**Figure 4:**
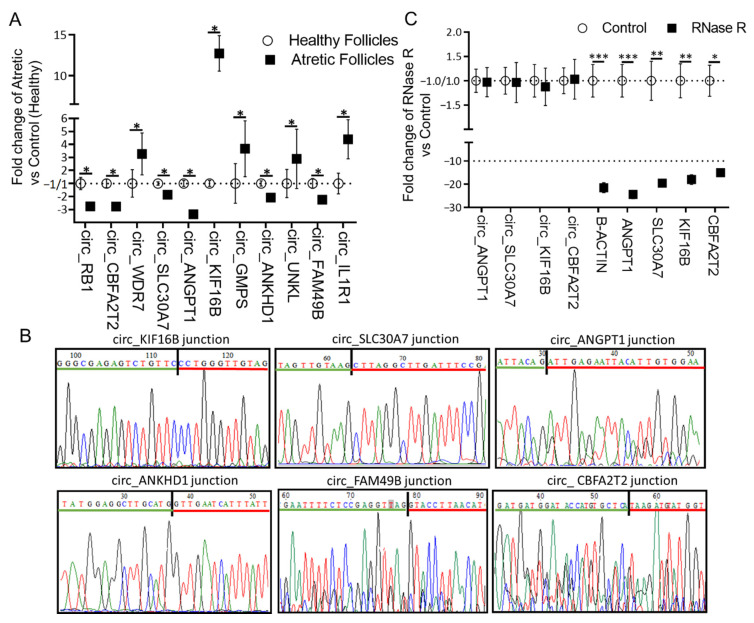
Validation and characterization of circRNAs. (**A**) Relative circRNA expression measured by qRT-PCR. circRNA expression was calculated as a fold change of AA over HA follicles. circRNA expression in HA was set to 1 for up-regulated genes and −1 for down-regulated genes. (**B**) Representative examples of PCR products purified and sequenced to confirm circRNA junction sites by Sanger sequencing, *n* = 3. (**C**) qRT-PCR for the abundance of circRNAs (*n* = 6) and mRNAs (*n* = 6) treated with RNase R. The amount of circRNAs and mRNAs was calculated as fold change of RNase R over controls. The expression values in the controls were set to 1 for up-regulated genes and −1 for down-regulated genes. *, *p* < 0.05; **, *p* < 0.01; ***, *p* < 0.001. Follicles were isolated in batches at three different time points, each batch containing 15–20 ovaries.

Outward-facing divergent primers (Table S3) were designed against 10 randomly selected DE-circRNAs, and their expression levels were measured by qRT-PCR (Figure 4A). The expression levels of circ_RB1, circ_CBFA2T2, circ_SLC30A7, circ_ANGPT1, circ_ANKHD1, and circ_FAM49B were significantly lower, while the expression levels of circ_WDR7, circ_KIF16B, circ_GMPS, circ_UNKL, and circ_IL1R1 were significantly higher in granulosa cells of AA follicles compared to HA follicles, confirming the RNA-seq data. In addition, we further confirmed the correctness of the amplified PCR products with specific circRNA junctions for circ_KIF16B, circ_SLC30A7, circ_ANGPT1, circ_ANKHD1, circ_FAM49B, and circ_CBFA2T2 by Sanger sequencing (Figure 4B).

Four circRNAs (circ_ANGPT1, circ_SLC30A7, circ_KIF16B, and circ_CBFA2T2) and four corresponding linear mRNAs (ANGPT1, SLC30A7, KIF16B, and CBFA2T2) serving as controls were randomly selected for RNase R resistance analysis (Figure 4C). The qRT-PCR results showed that the expression levels of these four circRNAs following RNase R digestion were similar to the untreated controls, while the expression levels of all four liner mRNAs were significantly decreased after RNase R treatment. These results further confirmed that these circRNAs were indeed circular in form.

### 2.6. circRNA-miRNA Interaction Network

A circRNAs-miRNA interaction network was constructed using Cytoscape based on our RNA-seq data (Figure S4). The top 10 up-regulated circRNAs and the top 20 down-regulated circRNAs were selected to identify circRNA-miRNA interactions based on TargetScan and miRanda analysis. The top five miRNAs for each circRNA were selected based on their context+ score.

When applying our stringent selection criteria for candidate circRNAs, two exonic circRNAs, with miRNA binding sites and fulfilling all criteria, namely, circ_CBFA2T2 (FC = 13; FDR = 0.007, being one of the down-regulated circRNAs) and circ_KIF16B (FC = 13.5; FDR = 0.004, being one of the most up-regulated circRNAs) (Table 1), attracted our interest. Following KEGG pathway analysis of the predicted targeted genes of circ_CBFA2T2 (Figure S5A), we observed that the top enriched process referred to response to oxidative stress, a key trigger of apoptosis in granulosa cells [26]. Similarly, the pathway analysis of putatively targeted genes of circ_KIF16B showed that the main enriched processes directly associated with ovarian follicular development were the apoptosis-related pathways, including the phosphatidylinositol 3-kinase (PI3K)-protein kinase B (AKT) athway and apoptosis pathways (Figure S5B). These two circRNAs were, therefore, selected for further functional analysis.

### 2.7. The Putative Role of Circ_CBFA2T2 in Apoptosis of Granulosa Cells in Antral Follicles

**Figure 5:**
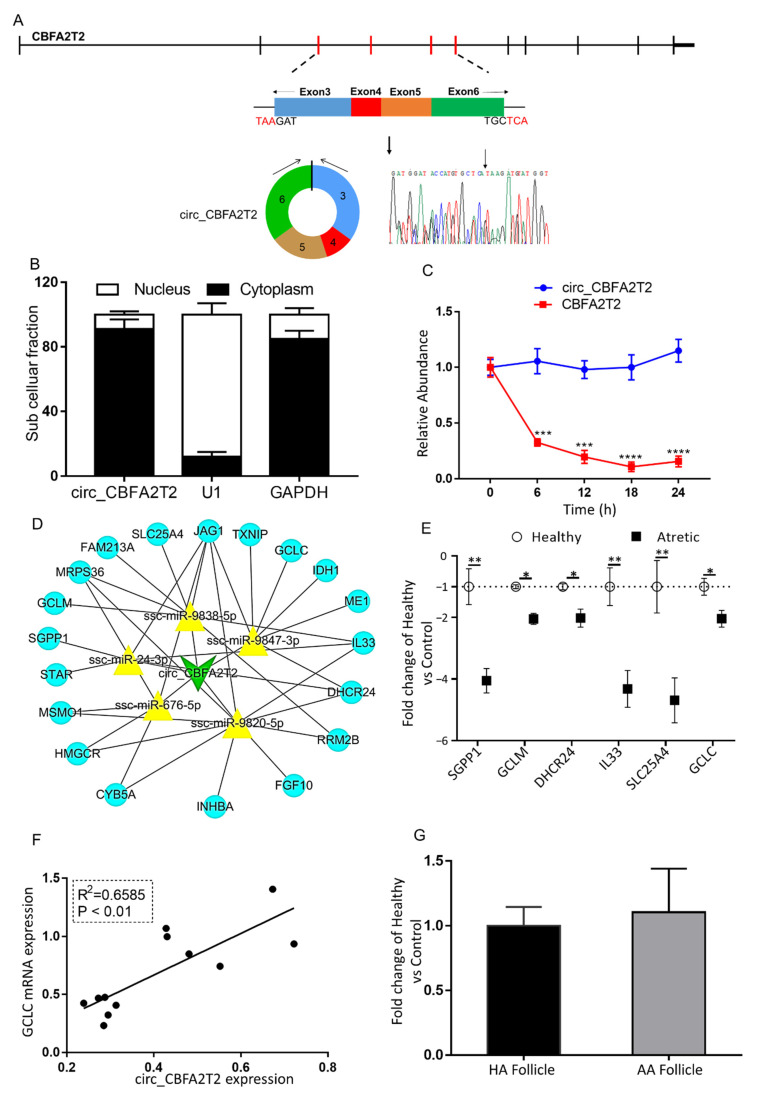
The characterization and putative role of circ_CBFA2T2. (**A**) The genomic loci of circ_CBFA2T2 in the *CBFA2T2* gene. The expression of circ_CBFA2T2 was validated by qRT-PCR, followed by Sanger sequencing. Arrows represent divergent primers binding to the genome region of circ_CBFA2T2. (**B**) Cellular characterization of circ_CBFA2T2. Levels of nuclear control transcript (U1), cytoplasmic control transcript (GAPDH and mRNA), and circ_CBFA2T2 were assessed by qRT-PCR in nuclear (*n* = 3) and cytoplasmic (*n* = 3) fractions of porcine granulosa cells. Data are presented as a percentage of U1, GAPDH, and circ_CBFA2T2 levels. (**C**) qRT-PCR for the abundance of circ_CBFA2T2 (blue line, *n* = 3) and *CBFA2T2* mRNA (red line, *n* = 3) in granulosa cells treated with actinomycin D at the indicated time points. (**D**) A putative ceRNA network of circ_CBFA2T2 constructed by Cytoscape 3.8. The green V shape represents circ_CBFA2T2, the yellow triangle represents targeted miRNAs, and blue circles denote targeted mRNAs. (**E**) qRT-PCR for the abundance of mRNA content of eight targeted genes in granulosa cells of HA (open circles, *n* = 6) and AA (filled squares, *n* = 6) follicles. (**F**) Association of expression levels of circ_CBFA2T2 and *GCLC*. (**G**) circ_CBFA2T2 expression in cumulus cells of HA (black bar, *n* = 6) and AA (grey bar, *n* = 6) follicles, as measured by qRT-PCR. Gene expression, as fold change of AA over HA follicles, with no change, was indicated as 1. *, *p* < 0.05; **, *p* < 0.01. Follicles were isolated in batches at three different time points, each batch containing 15–20 ovaries.

Circ_CBFA2T2 was derived from four exons, including exon 3–6 of the gene *CBFA2T2* (Figure 5A). qRT-PCR analysis of nuclear and cytoplasmic expression demonstrated that circ_CBFA2T2 was mainly localized in the cytoplasm of granulosa cells (Figure 5B). The stability of circ_CBFA2T2 was investigated in granulosa cells treated with actinomycin D, an inhibitor of transcription. Total RNA was harvested at the indicated time points after treatment with actinomycin D (Figure 5C). Analysis of circ_CBFA2T2 and *CBFA2T2* mRNA showed that circular transcript half-life exceeded 24 h, whereas the associated linear transcript exhibited a half-life of less than 6 h, indicating that the circ_CBFA2T2 was highly stable.

The circ_CBFA2T2-miRNA-mRNA network was constructed using Cytoscape to visualize interactions (Figure 5D). The top five most likely sponged miRNAs (ssc-miR-9847-3p, ssc-miR-9838-5p, ssc-miR-24-3p, ssc-miR-676-5p, ssc-miR-9820-5p) and 20 representative targeted genes of these miRNAs were identified. In line with the expression of circ_CBFA2T2, mRNA content of two targeted apoptosis-related genes—sphingosine-1-phosphate phosphatase 1 (*SGPP1*) and interleukin 33 (*IL33*)—as well as four genes related to oxidative stress and mitochondrial functioning, namely, glutamate-cysteine ligase (*GCLM*), 24-dehydrocholesterol reductase (*DHCR24*), solute carrier family 25 member 4 (*SLC25A4*), and glutamate-cysteine ligase catalytic subunit (*GCLC*), was significantly down-regulated in granulosa cells of AA follicles compared to granulosa cells of HA follicles (Figure 5E).

Interestingly, these analyses showed that expression of circ_CBFA2T2 was significantly associated with the mRNA content of *GCLC*, a key antioxidant gene controlled by ssc-miR-9847-3p (Figure 5F). The transcript content of circ_CBFA2T2 in the cumulus cells of AA follicles was similar to that of cumulus cells in HA follicles (Figure 5G).

### 2.8. The Putative Role of Circ_KIF16B in Apoptosis of Granulosa Cells in Antral Follicles

**Figure 6:**
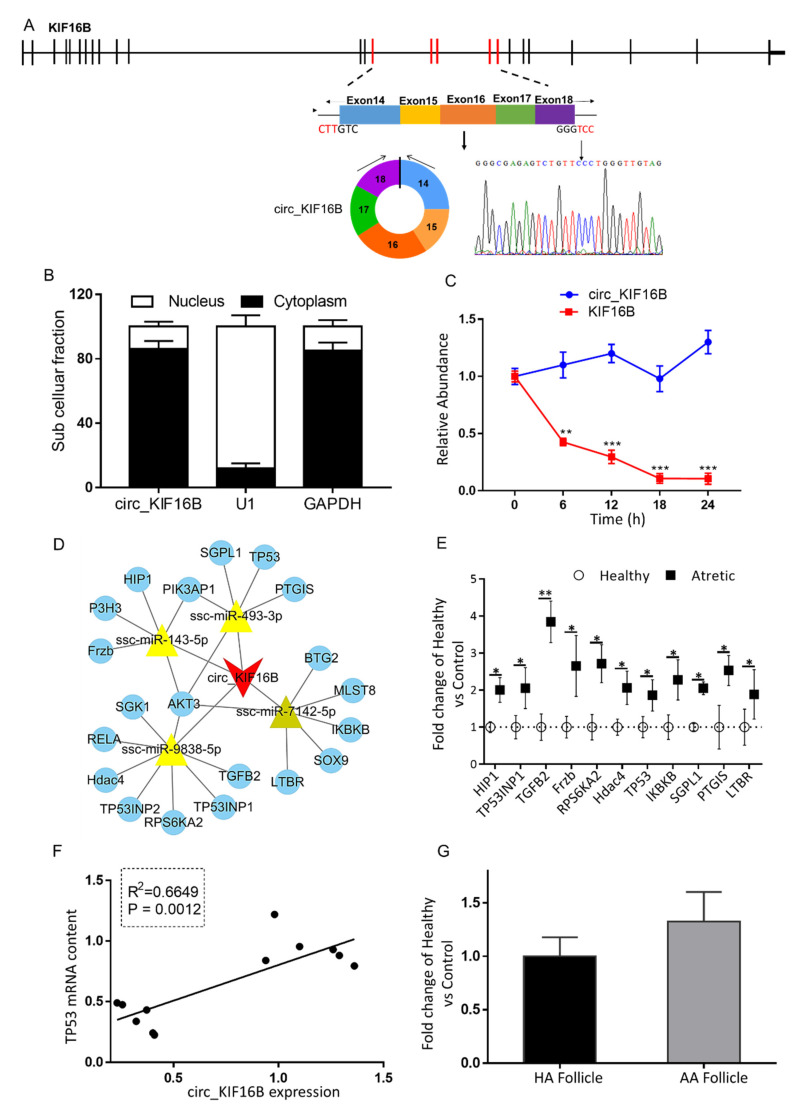
Characterization and putative role of circ_KIF16B. (**A**) The genomic loci of circ_KIF16B in the *KIF16B* gene. The expression of circ_KIF16B was validated by qRT-PCR, followed by Sanger sequencing. Arrows represent divergent primers binding to the genome region of circ_KIF16B. (**B**) Cellular characterization of circ_KIF16B. Levels of nuclear control transcript (U1), cytoplasmic control transcript (GAPDH and mRNA), and circ_KIF16B were assessed by qRT-PCR in nuclear (*n* = 3) and cytoplasmic fractions (*n* = 3) of porcine granulosa cells. Data are presented as a percentage of U1, GAPDH, and circ_KIF16B levels. (**C**) qRT-PCR for the abundance of circ_KIF16B (blue line, *n* = 3) and KIF16B mRNA (red line, *n* = 3) in granulosa cells treated with actinomycin D at the indicated time points. (**D**) A putative ceRNA network of circ_KIF16B constructed by Cytoscape 3.8. The red V shape represents circ_KIF16B, the yellow triangle represents targeted miRNAs, and blue round circles denote targeted mRNAs. (**E**) qRT-PCR for the abundance of mRNA content of 10 targeted pro-apoptotic genes in granulosa cells of HA (open circles, *n* = 6) and AA (filled squares, *n* = 6) follicles. (**F**) Correlation of expression levels of circ_KIF16B and *TP53*. (**G**) circ_KIF16B expression in cumulus cells of HA (black bar, *n* = 6) and AA follicles (grey bar, *n* = 6), as measured by qRT-PCR. Gene expression, as fold change of AA over HA follicles, with no change, was indicated as 1. *KIF16B*, kinesin family member 16B; *, *p* < 0.05; **, *p* < 0.01. Follicles were isolated in batches at three different time points, each batch containing 15–20 ovaries.

Circ_KIF16B was derived from five exons, including exon14–18 of the *KIF16B* gene (Figure 6A). Besides circ_KIF16B, there were four other circular transcripts observed that were derived from the *KIF16B* gene (named circ_KIF16B2, circ_KIF16B3, circ_KIF16B4, circ_KIF16B5, respectively). Circ_KIF16B was the predominant circular isoform as this isoform had the highest number of back-spliced unique reads (circ_KIF16B, 54; circ_KIF16B2, 26; circ_KIF16B3, 6; circ_KIF16B4, 9; circ_KIF16B5, 12). The qRT-PCR analysis of circ_KIF16B expression demonstrated that the circular form of KIF16B preferentially localized in the cytoplasm (Figure 6B). Similar to circ_CBFA2T2, the stability of circ_KIF16B was investigated using actinomycin D (Figure 6C). The analysis of circ_KIF16B and *KIF16B* mRNA showed that the circular transcript half-life exceeded 24 h, whereas the associated linear transcript exhibited a half-life of less than 6 h, indicating that the expression of circ_KIF16B was also highly stable.

CircRNA-miRNA-targeted mRNA network analysis revealed the identification of the top four most likely sponged miRNAs (ssc-miR-493-3p, ssc-miR-9838-5p, ssc-miR-7142-5p, ssc-miR-143-5p) and 20 representative targeted genes of these miRNAs (Figure 6D). In order to detect whether the expression of the targeted mRNAs was associated with circ_KIF16B expression, the qRT-PCR was used to measure mRNA content of 10 targeted pro-apoptotic related genes, namely huntingtin interacting protein 1 (*HIP1*), tumor protein p53 inducible nuclear protein 1 (*TP53INP1*), transforming growth factor-beta 2 (*TGFB2*), frizzled-related protein (*Frzb*), ribosomal protein S6 kinase A2 (*RPS6KA2*), tumor protein p53 (*TP53*), an inhibitor of kappa light polypeptide gene enhancer in B-cells kinase beta (*IKBKB*), sphingosine-1-phosphate lyase 1 (*SGPL1*), prostaglandin I2 synthase (*PTGIS*), and lymphotoxin beta receptor (*LTBR*). In line with the expression of circ_KIF16B, the mRNA content of these 10 genes was significantly up-regulated in granulosa cells of AA follicles (Figure 6E).

**Figure 7:**
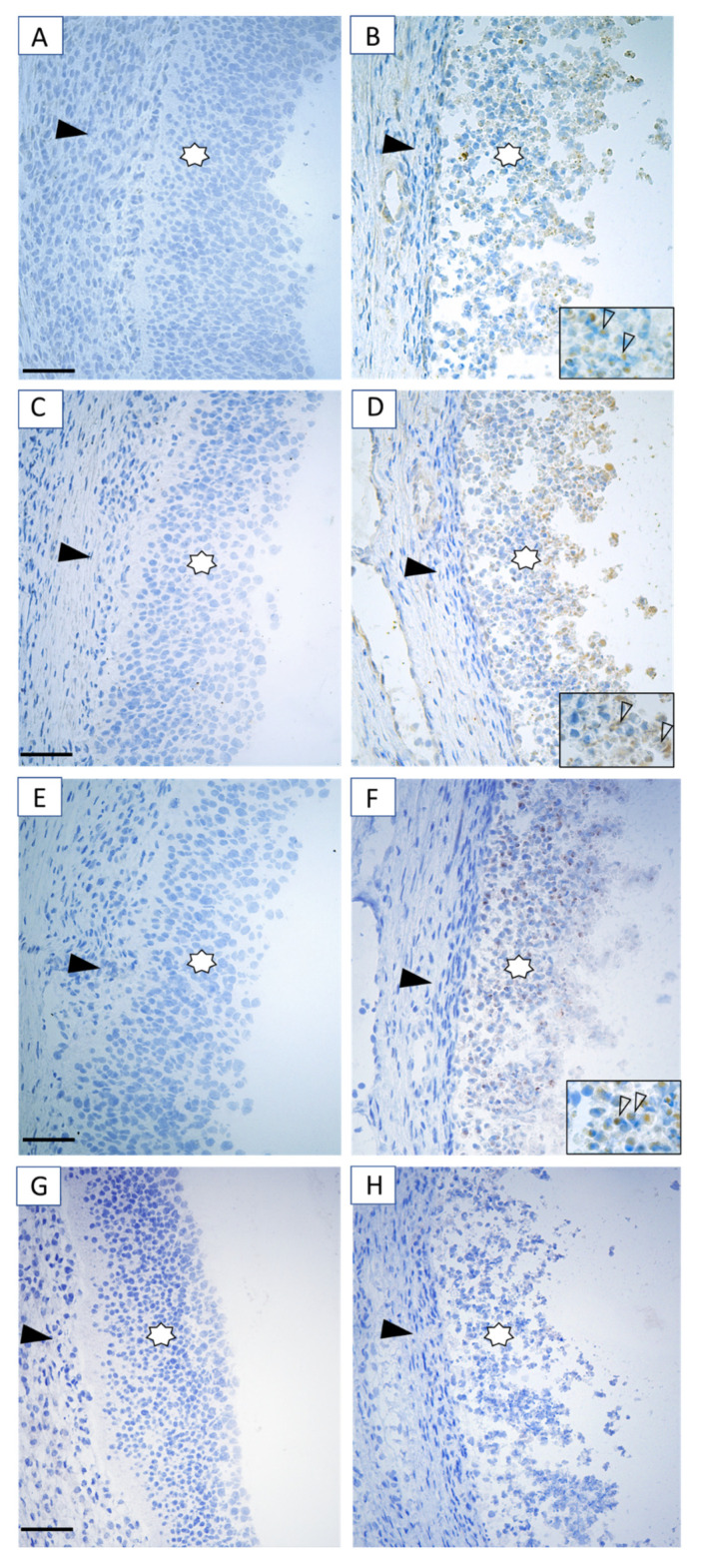
Immunostaining for the presence of p53, PHLDA3, and cCASP3 (brown staining) as markers of the p53 signaling pathway and apoptosis in the HA (**A**,**C**,**E**) and AA (**B**,**D**,**F**) follicles (*n* = 6, ovaries). (**A**,**B**) Representative immunostaining of p53, (**C**,**D**) PHLDA3, (**E**,**F**) cCASP3, and (**G**,**H**) negative controls. Immunostaining of p53, PHLDA3, and cCASP3 was faint to absent in granulosa cells of HA follicle, but moderate to strong in granulosa cells of AA follicles. Staining was faint to absent in theca cells. Insets show a detail of the granulosa layer. Granulosa cells are indicated by asterisks, theca cells by an arrowhead. Scale bars represent 50 µm. HA, healthy antral follicles; AA, atretic antral follicles. cCASP3, cleaved caspase 3.

To determine whether the up-regulation of circ_KIF16B expression was associated with the expression of the above 10 targeted mRNAs, linear regression analyses were performed. Interestingly, these analyses showed that the expression of circ_KIF16B was significantly associated with the mRNA content of *TP53*, a hub gene controlling apoptosis suppressed by ssc-miR-493-3p (Figure 6F). In line with mRNA expression data, immunohistochemical staining for p53 in granulosa cells of HA follicles was weak to absent (Figure 7A). In contrast, moderate to strong staining was observed in both the nuclei and cytoplasm of apoptotic granulosa cells in antral follicles (Figure 7B), indicating the activation of p53 signaling during granulosa cell apoptosis.

Next, the expression of circ_KIF16B in cumulus granulosa cells of HA and AA follicles was investigated. qRT-PCR results showed that the transcript content of circ_KIF16B in cumulus granulosa cells of AA follicles did not differ significantly from that of HA follicles, although there was an increasing trend (Figure 6G).

These results suggested that circ_KIF16B might promote apoptosis of granulosa cells, possibly by binding ssc-miR-493-3p, resulting in the up-regulation of the pro-apoptotic *TP53* gene.

### 2.9. p53/PHLDA3 Pathway and Antral Follicular Atresia

To further confirm the role of the p53 signaling pathway as the downstream target of circ_KIF16B in granulosa cells, qRT-PCR and immunohistochemical staining experiments were performed. Firstly, downstream targets of *TP53* were identified. qRT-PCR was used to measure the mRNA content of B-cell lymphoma **2** binding component 3 (*PUMA)*, phorbol-12-myristate-13-acetate-induced protein 1 (*BIM*), and pleckstrin homology like domain family a member 3 (*PHLDA3*) in the granulosa cells of HA and AA follicles. These genes were selected based on the fact that they are reported p53 target genes in other cell types, including tumor cells and fibroblast cells [27,28]. Only the mRNA content of *PHLDA3* was increased by more than five times in AA follicles compared to HA follicles, indicative of being a possible target of p53 in apoptotic granulosa cells. Immunohistochemical staining was used to detect the protein presence of p53, PHLDA3, and cCASP3 in the same HA and AA follicles. Consistent with the gene expression of *PHLDA3*, the immunostaining for PHLDA3 in granulosa cells of HA follicles was faint to absent (Figure 7C), while moderate to strong staining was observed in the apoptotic granulosa cells of AA follicles (Figure 7D). Similarly, cCASP3 staining was absent in the granulosa cells of HA follicles (Figure 7E); however, many apoptotic cells with cCASP3 positive staining were present in the granulosa cell layer of AA follicles (Figure 7F).

## 3. Discussion

This study was to our knowledge the first investigation that compared circRNAs profiles in granulosa cells of HA and advanced stage AA follicles in the porcine ovary. A large number of circRNAs from diverse genomic locations were identified; 62 of these circRNAs were DE-circRNAs. KEGG analysis of the host genes of the DE-circRNAs showed that apoptosis was one of the significantly enriched pathways. The characteristics of the DE-circRNAs, including back-splicing, RNase R resistance, and stability, were validated and suggested that these transcripts were real circular in form. We further showed that *circ_CBFA2T2* and *circ_KIF16B* were two of the most relevant down-regulated and up-regulated exonic DE-circRNAs with miRNA binding sites. We further confirmed the expression of the targeted genes of these circRNAs, the antioxidant gene *GCLC* (potential target of circ_CBFA2T2), and the apoptotic gene *TP53* (potential target of circ_KIF16B). Lastly, we showed that the p53/PHLDA3 pathway might participate in granulosa cell apoptosis. Based on these results, we hypothesized that these circRNAs might potentially play a role in granulosa cells apoptosis and thus antral follicular atresia.

Interestingly, despite relatively low expression levels per circRNA species, the diversity of the observed circRNA species is high, and many circRNA isoforms can be expressed by a single gene [29]. In line with this observation, our results showed that one parental gene could produce several circRNA isoforms, sometimes even more than 10. Only one or two isoforms were usually expressed at dominant levels, while most other isoforms exhibited low expression levels, suggesting that the dominant form might be purposefully produced to serve a specific function in granulosa cells.

CircRNAs have been suggested to function as miRNA sponges and participate in miRNA-mediated post-transcriptional regulation. Only a limited number of such circRNAs, however, contain multiple binding sites to sequester a particular miRNA. For example, circRNA—ciRS-7 harbors more than 70 conserved binding sites and acts as a sponge for miR-7 to regulate brain cell development [30]. Accumulating evidence suggests, however, that these large numbers of putative targeting sites are not necessary for circRNA to act as a miRNA sponge. An abundant circRNA derived from exon2 of the *HIPK3* gene harbors only two binding sites for miR-124, but can despite this low number of targeting sites directly inhibit the activity of miR-124 [23]. In line with these observations, the present study showed that most of the DE-circRNAs harbored only one to two targeting sites for miRNAs. For instance, circ_CBFA2T2 and circ_KIF16B, located mainly in the cellular cytoplasm, had only two putative binding sites for ssc-miR-9847-3p and one putative binding site for ssc-miR-493-3p, respectively.

Several studies have reported that oxidative stress caused by the presence of the xenotoxic stressors can lead to ovarian follicular atresia [6,26]. In the present investigation, the mRNA concentrations of *GCLC*, the putative targeted gene of circ_CBFA2T2, and *GCLM* were significantly lower in the AA follicles compared to HA follicles. *GCLC* and *GCLM* are two subunits of glutamate-cysteine ligase, which acts as a rate-limiting enzyme in glutathione synthesis, an important antioxidant [31]. Hatzirodos et al. [22] observed a six-fold decrease in *GCLC* mRNA concentrations in small AA follicles in the bovine ovary. These observations strengthen the assumption of a disturbed glutathione antioxidant capacity in antral follicular atresia. Based on these observations, we speculated that circ_CBFA2T2 might play a role in antral follicular atresia via targeting *GCLC*.

miRNAs play an important role in the regulation of apoptotic processes. Kleemann et al. showed in ovarian carcinoma cell lines that miR-493-3p mimic transfection led to the disruption of the mitochondrial membrane potential and activation of caspases and apoptosis [8]. ssc-miR-493-3p is predicted to bind to circ_KIF16B, one of the identified DE-circRNAs in the present study. A predicted targeted gene of ssc-miR-493-3p is *TP53,* a gene in which the mRNA content was significantly up-regulated in granulosa cells of AA follicles in our study. The up-regulation of *TP53* mRNA content coincided with intense p53 immunostaining in apoptotic granulosa cells of AA follicles, implicating that *TP53* gene translation occurred, while p53 immunostaining was faint to absent in granulosa cells of HA follicles. This p53 immunostaining pattern coincided with previous observations in the rat ovary [32].

Recently, Haraguchi et al. (2019) found that in murine ovarian cumulus granulosa cells, the mouse double minute 2 homolog (Mdm2) - steroidogenic factor 1 (SF1) pathway played an important role in oocyte maturation by suppressing p53 activation; deletion of *Mdm2* gene expression led to the activation of p53 [33]. We, however, did not observe differences in the expression of *Mdm2* and *SF1* in mural granulosa cells of healthy and atretic antral follicles (data not shown). Our data thus suggested that the activation of p53 in mural granulosa cells of atretic antral follicles in the porcine ovary did not seem to be due to a decrease *in Mdm2* and *SF1* gene expression.

It has been reported for other cell types, including fibroblasts and skin keratinocytes, that p53 preferably induces apoptosis via the B-cell lymphoma 2 (BCL-2)-regulated intrinsic apoptotic pathway by directly binding to its target genes, such as *PUMA* and *BIM* [27]. In our study, granulosa cells apoptosis, however, did not seem to favor these target genes as the mRNA content of *BIM* and *PUMA* was not altered in AA follicles compared to HA follicles. This suggested that another target gene must be involved in the p53-dependent apoptotic pathway in granulosa cells. Recently, a novel target gene of p53, named *PHLDA3*, has been shown to induce apoptosis by inhibiting the activity of the PI3K-Akt pathway in tumor cells [28,34]. The PI3K-Akt pathway plays an essential role in granulosa cell growth and apoptosis [5]. PHLDA3, a PH domain-only protein, can repress Akt activity by competitively binding to phosphatidylinositol 4,5-bisphosphate (PIP)2 and PIP3, leading to the activation of caspases and apoptosis. Ablation of endogenous PHLDA3 results in enhanced Akt activity and a decrease of p53-dependent apoptosis [28]. Our results showed that the mRNA content of *PHLDA3* was significantly increased in AA follicles compared to HA follicles. Concomitantly, clear immunostaining of PHLDA3 and cCASP3 was confined to apoptotic granulosa cells in AA follicles, whereas staining in the granulosa cells of HA follicles was faint to absent. This suggested a potential role for PHLDA3 in granulosa cell apoptosis. We, therefore, hypothesized that circ_KIF16B might be involved in antral follicle atresia by serving as a miRNA sponge, which sequesters ssc-miR-493-3p, activating the p53/PHLDA3 signaling pathway and thus promoting granulosa cell apoptosis. The underlying molecular mechanisms of circ_CBFA2T2 and circ_KIF16B in granulosa cell apoptosis, however, needs to be further investigated.

Investigations of the role of circRNAs in ovarian follicular development so far have mainly focused on either the physiology of antral follicular growth or on ovarian diseases, e.g., PCOS. Not much is known about the role of circRNAs in follicle atresia. In a recent mouse study conducted by Jia and colleagues, it is reported that circRNAs might participate in ovarian granulosa cell apoptosis [12]. RNA-Seq analysis of pubertal (six-week-old) and neonatal (5-day-old) mouse ovaries showed up-regulation of circ_EGFR in the pubertal ovary. In vitro overexpression of circ_EGFR promoted the proliferation of granulosa cells, while knockdown of circ_EGFR significantly decreased the rate of proliferation, possibly via increased granulosa cell apoptosis. Our observations from the in vivo perspective extended these findings, as we identified by RNA-Seq 62 DE-circRNAs from a total of 9632 circRNAs in isolated porcine granulosa cells of HA and AA follicles. KEGG pathway analysis of the host genes of these DE-circRNAs showed that the apoptosis pathway was significantly enriched. We observed an increased mRNA content and strong immunostaining of p53/PHLDA3, a target of circ_KIF16B, in granulosa cells of AA follicles compared to HA follicles. These results offered further support for the role of circRNAs in the apoptosis of ovarian granulosa cells. Apart from our study, Guo et al. [35] showed that circ_INHA, generated from the intron of the inhibin subunit alpha gene, was down-regulated in porcine granulosa cells of early AA follicles compared to healthy follicles. circ_INHA inhibited granulosa cell apoptosis, possibly through sponging miR-10a-5p, thus modulating connective tissue growth factor (CTGF, CNN2) expression. CTGF, a member of the calponin (CNN) matricellular protein family, plays a role in many biological processes, including cell proliferation and apoptosis [35]. In contrast to the study by Guo and colleagues, we did not observe differential expression of circ_INHA via deep circRNA-Seq in granulosa cells from HA and advanced stage AA follicles. This discrepancy might be due to differences in the size of the selected antral follicles, as the present study analyzed DE-circRNAs in freshly isolated granulosa cells from 4-7 mm healthy and advanced atretic antral follicles collected during the follicular phase of the estrous cycle, while Guo and colleagues used somewhat smaller 3–5 mm antral follicles and analyzed DE-circRNAs in granulosa cells of healthy and early atretic antral follicles collected at an unknown stage of the estrous cycle.

In ovarian antral follicles, granulosa cells are subdivided into two distinct classes that are anatomically and functionally distinct: mural granulosa cells, which line the follicular wall, and cumulus granulosa cells, which surround and are in direct contact with the oocyte, together forming the cumulus-oocyte-complex (COC) [36]. There are several observations that have demonstrated that in antral follicular atresia, mural granulosa cells undergo apoptosis, while, at the same time, the cumulus cells remain healthy [37,38]. We confirmed these observations as we did not see morphological differences between COCs retrieved from AA or HA follicles. The absence of a difference in transcript content of circ_CBFA2T2 and circ_KIF16B between cumulus granulosa cells from HA and AA follicles further supported the morphological observation that during follicular atresia when increasing numbers of mural granulosa cells underwent apoptosis, cumulus cells remained healthy. It is not until late-stage atresia when most mural cells have undergone apoptosis that the cumulus cells also become apoptotic [36].

## 4. Materials and Methods

### 4.1. Chemicals

All chemicals were purchased from Sigma (Guangzhou, China) unless indicated otherwise. Antibodies against p53 (lot no. GR316086-42, cat. no. ab131442) and pleckstrin homology like domain family A member 3 (PHLDA3, lot no. GR3295493-2, cat. no. ab196757) were purchased from Abcam (Cambridge, UK). The antibody against cleaved caspase 3 (cCASP3, lot no 45, cat. no. 96615) was purchased from Cell Signaling Technology (Leiden, The Netherlands).

### 4.2. Animal and Follicles Collection

**Figure 8:**
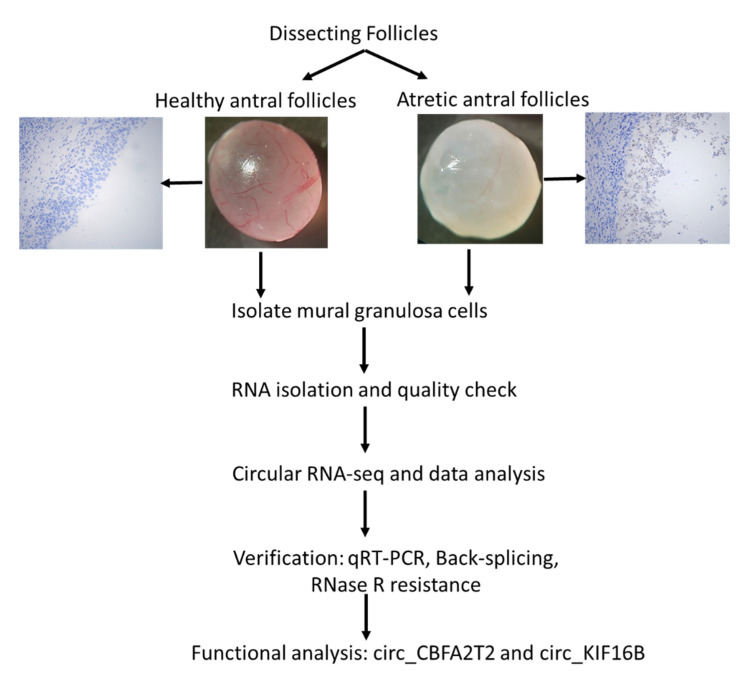
Overview of experimental design.

The whole experiment design was depicted as shown in the Figure 8. Sixty porcine ovaries from 30 gilts (nulliparous, around 180 days old with a bodyweight of approximately 120 kg) were used in this study. The gilts were housed at a local farm and monitored once daily to determine the stage of the estrous cycle in the presence of a boar. The first day of the estrous cycle onset was counted as day 0 of the estrous cycle. The animals were slaughtered in a local abattoir on day 16–19 of the estrous cycle, the beginning to mid follicular phase of the cycle [39]. In total, 60 ovaries were collected in our study, of which six ovaries from six different animals were snap-frozen immediately after the slaughter in the nitrogen liquid and stored at −80 °C until further immunohistochemical processing. The other 54 ovaries were collected immediately after slaughter and washed in 0.01 M phosphate-buffered saline pH 7.4 (PBS), transferred to a 30 °C PBS solution containing 1% penicillin-streptomycin, and transported to the laboratory within 1 h after slaughter. To ensure that follicle dissection was finished within a reasonably short time frame, the sows were slaughtered at three different days (three different batches). At each slaughter day, 15 to 20 ovaries were collected for antral follicle dissection (in total, 54 ovaries were used for follicle dissection). While dissecting one ovary, the remaining ovaries of that batch were stored in dulbecco’s phosphate buffered saline (DPBS) on ice. For each batch, follicles were dissected within 1.5 h after arrival in the lab. Antral follicle size was estimated by measurement of two perpendicular diameters with a millimeter scale. Individual antral follicles, approximately 4–7 mm in diameter, were dissected from the ovaries.

Healthy antral (HA) follicles and atretic antral (AA) follicles were classified morphologically under a stereomicroscope, as described previously [40,41,42]. Briefly, HA follicles were characterized by a vascular sheath on the follicular surface with a pinkish color and clear follicular fluid in the antrum. AA follicles were identified by an opaque color, the absence of clear vascularization on the surface of the follicle, and a large amount of debris floating in the follicular fluid [42,43]. Based on the criteria from Gioia et al. [43], these AA follicles were considered to be at an advanced stage of atresia.

In order to confirm the classification, 10 randomly selected follicles classified as either well-vascularized or non-vascularized from total 245 dissected follicles were fixed in diluted Bouin’s fluid (0.9% picric acid, 4% formaldehyde, 5% glacial acetic acid) for 24 h at 4 °C and embedded in paraffin. The follicles were sectioned at a thickness of 5 μm. Six sections of each follicle were randomly selected, mounted on glass slides, stained with periodic acid Schiff’s reagent (PAS) and Mayer’s hematoxylin, and examined by light microscopy. Antral follicles were considered to be atretic when more than 5% of the granulosa cells showed signs of apoptosis, while the theca layer of these follicles showed signs of hypertrophy. In more advanced atretic follicles, apoptotic granulosa cells were not only observed in the mural cell layer but also throughout the follicular antrum [5,26]. The status of follicular atresia was further confirmed by immunostaining for the presence of cCASP3 (see below). The histological analysis confirmed the macroscopical identification of HA and AA follicles (Figure S1).

Based on the criteria described above, 120 follicles (60 HA follicles and 60 advanced AA follicles) were selected from a pool of 235 follicles; the 115 follicles that were not included in the follicle selection did not meet our selection criteria for healthy and advanced atretic antral follicles based and were thus excluded from sampling. These two pools of 60 follicles were randomly divided into 6 groups of 10 follicles each. Follicular fluid was collected and pooled per 10 follicles. In the advanced stage atretic antral follicles, apoptotic granulosa cells had become lose from the follicle wall and were floating in the antrum, mixing with the follicular fluid. When dissecting these follicles, the follicular fluid and floating apoptotic granulosa cells were collected together in a Petri dish. The mural granulosa cells of these atretic follicles were scraped from the follicular wall and mixed with this follicular fluid to obtain a complete sample of granulosa cells (floating and mural cells) from the atretic follicles. To create comparable granulosa cell samples, the mural granulosa cells scraped from the walls of healthy antral follicles were also mixed with the follicular fluid. In brief, follicles were dissected in a Petri dish in PBS and cut in smaller segments. Follicular wall fragments (consisting of theca and mural granulosa layer) and the cumulus-oocyte-complex (COC) were separated. COCs were collected with a Pasteur pipette and transferred to another Petri dish, where cumulus granulosa cells were mechanically stripped from the oocyte. The collected cumulus cells were washed in PBS, centrifuged, and then stored at −80 °C until RNA extraction. Simultaneously, the mural granulosa cells were scraped off from the dissected follicle wall by gentle rubbing with a glass round-smooth Pasteur pipette [22]. The scraped granulosa cells were mixed with the follicular fluid of the corresponding follicles, diluted 1:3 in PBS, transferred to a 1.5 mL Eppendorf (EP) tube, and centrifuged at 800 g for 5 min. The supernatant was collected and stored at −80 °C for subsequent hormone analysis. The residual granulosa cells remaining in the precipitate were stored at −80 °C until RNA isolation.

To test the purity of the isolated granulosa cells, cells were labeled with an antibody against the follicle-stimulating hormone receptor (FSHR, ab150557, Abcam), a specific marker for granulosa cells [44]. On average, approximately 1 × 10^4^ cells in three biological replicates (three different pools of follicles derived from three different batches of sows) were analyzed. After FSHR immunostaining, pictures were taken by a light microscope (Leica DM6000B, equipped with a DFC356FX digital camera and LasX imaging software; Leica Microsystems, Guangzhou, China) and analyzed using Image J software.

### 4.3. Granulosa Cells Cultured In Vitro

To test the stability of circRNAs, in vitro cultured granulosa cells were treated with actinomycin D, a transcription inhibitor. Isolated granulosa cells were seeded into 6-well or 12-well plates and cultured in Dulbecco’s Modified Eagle Medium/Nutrient Mixture F-12 supplied with 10% FBS and 1% penicillin-streptomycin (Gibco, Thermo Fisher, Shanghai, China) for 24 h. Transcription was blocked by adding 2 µg/mL actinomycin D or dimethylsulfoxide (Sigma-Aldrich, St. Louis, MO, USA) as a control, with three replicates for each treatment under five time-points of 0, 6, 12, 18, and 24 h, respectively. At each time point, cells were collected for RNA isolation, and qRT-PCR was used to quantify the content of circRNAs and their linear mRNAs (see below).

### 4.4. Follicle Fluid IGF1, AMH, and Estradiol Measurements

Insulin-like growth factor 1 (IGF1, follicle fluid diluted 1:3; Porcine IGF1 ELISA Kit; CUSABIO, Wuhan, China), anti-Müllerian hormone (AMH, follicle fluid diluted 1:4; Porcine AMH ELISA Kit; CUSABIO), and estradiol levels (follicle fluid diluted 1:2; Porcine estradiol ELISA Kit, Enzo Life Sciences, Shenzhen, China) were determined, according to the protocol of the respective suppliers as reported previously [45]. The inter-assay variation was less than 10% for all assays.

### 4.5. Circular RNA Library Construction and Illumina Sequencing

Total RNA from granulosa cells of three randomly selected samples of pooled granulosa cells from either 10 HA or 10 AA follicles was isolated using TRIzol (Life Technologies, Shanghai, China) for the purpose of RNA-sequencing (RNA-seq). High throughput transcriptome sequencing was carried out by Cloud-Seq Biotech (Shanghai, China). Briefly, total RNA extraction and rRNA depletion were performed using the Ribo-Zero rRNA Removal Kit (Illumina, Shanghai, China), according to the manufacturer’s instructions. RNA libraries were constructed from the rRNA-depleted RNA using the TruSeq Stranded Total RNA Library Prep Kit (Illumina), according to the manufacturer’s instructions. The quality and quantity of the libraries were checked by the BioAnalyzer 2100 system (Agilent Technologies, Palo Alto, CA, USA). Ten pM libraries were denatured, captured on Illumina flow cells, amplified in situ, and finally sequenced for 150 cycles using the Illumina HiSeq Sequencer, according to the manufacturer’s instructions. All Illumina sequencing data have been submitted to the Gene Expression Omnibus (GEO) under accession number (GSE136589).

### 4.6. RNA-Seq Data Analysis

Data analysis was performed, according to Veno et al. [24], with minor modifications. Briefly, paired-end reads were harvested from the Illumina HiSeq 4000 sequencer; quality control was performed by Q30. After 3′adaptor-trimming, low-quality reads were removed using cutadapt software (v1.9.3, https://cutadapt.readthedocs.io/en/stable/) [46]. The remaining high-quality trimmed reads were used to analyze circRNAs.

The reads were aligned to the porcine genome (Sscrofa10.2/SusScr3) by employing bowtie2 software (v. 2.2.4, http://bowtie-bio.sourceforge.net/bowtie2/index.shtml) [47]. CircRNAs were detected and identified with find-circ software [30] downloaded from circBase [48]. The find-circ pipeline was run as suggested by the developers, except for an increased filtering stringency, requiring that both anchor segments mapped to the genome with mapping scores of 40. Only circRNAs, with two or more supporting reads within single samples, were used for further analysis.

Host genes, giving rise to individual circRNAs, were identified by matching the genomic location of circRNAs with the location of genes detected by TopHat/Cufflinks using BEDtools [49]. Published circRNAs were used to annotate the identified circRNAs. Back-splice junction reads for every circRNAs were normalized by mapped read numbers for each sample, and then log2 transformed. To detect differentially expressed circRNAs between granulosa cells from HA and AA follicles, a multiple testing correction method was used to estimate the false discovery rate (FDR). GO and pathway enrichment analysis were performed for the host genes of the differentially expressed circRNAs, using the Database for Annotation, Visualization, and Integrated Discovery (DAVID) v6.8 [50]. Differently expressed circRNAs were visualized as heatmaps using the MultiExperiment Viewer (MeV, http://mev.tm4.org/) [51].

### 4.7. miRNA Target Prediction and circRNA-miRNA-mRNA Network Construction

The predicted miRNA binding sites for the differentially expressed circRNAs were identified using the miRanda [52] and Targetscan [53] algorithms. Only those circRNA-miRNA interactions predicted by both algorithms were used for the downstream network construction and analyses. miRNA-mRNA interactions that were common in both miRanda and TargetScan were then used to determine the gene targets of each filtered miRNA. Using these data, the outline of a circRNA-miRNA-mRNA regulatory network was visualized using Cytoscape (version 3.8, https://cytoscape.org/).

### 4.8. Candidate circRNAs Selection

To confirm that these circRNAs indeed played a functional role via miRNA sponging in antral follicular atresia, candidate circRNAs were selected from differentially expressed (DE)-circRNAs according to the following criteria. To exert miRNA sponging function, these circRNAs needed to be stably located in the cytoplasm and have miRNA binding sites [23]. Thus exon-derived circRNAs, which were mainly cytoplasmic in location, should be suitable candidates for further investigation (Table 1) (criterium 1). Only DE-circRNAs with a fold change (FC) of at least 10 were considered to be suitable for selection (criterium 2). The DE-circRNAs should be consistently expressed in all samples with FDR values < 0.01 (criterium 3). Lastly, the functions of the target genes of the candidate circRNAs needed to be described in the literature and preferably should be directly associated with antral follicular atresia (criterium 4).

### 4.9. RNA Preparation and qRT-PCR

The nuclear and cytoplasmic fractions of three samples, each containing pooled granulosa cells of 10 HA and 10 AA follicles, were extracted using the PARIS KIT (Thermo Fisher, Shanghai, China) according to the manufacturer’s instructions. Briefly, approximately 10^7^ granulosa cells were collected, washed in PBS once, and then placed on ice. The granulosa cells were subsequently resuspended in 500 μL ice-cold cell fractionation buffer and incubated on ice for 10 min to induce complete lysis. The samples were centrifuged at 500× *g* for 5 min at 4 °C, after which the cytoplasmic fraction was carefully aspirated from the nuclear pellet. The nuclear fraction was further lysed in cell disruption buffer. Following this, total RNA from whole-cell lysates of the nuclear and cytoplasmic fraction, respectively, was isolated using TRIzol (Life Technologies).

Next, 3 μg of total RNA of each sample was incubated for 15 min at 37 °C with or without RNase R (3 U/μg RNA, Epicenter Technologies, Shanghai, China), followed by inactivation for 3 min at 95 °C. The resulting RNA was subsequently purified using the RNeasy MinElute Cleaning kit (Qiagen, Guangzhou, China). In order to guarantee similarly effective RNase R treatment, all RNA samples were treated simultaneously in one run.

To quantify the amount of circRNA and mRNA, the qRT-PCR analysis was performed; 1 μg RNA of each sample was used for cDNA synthesis using the PrimeScript RT Master Mix (Takara, Guangzhou, China). Quantitative RT-PCR reactions were performed employing SYBR Premix Ex Taq II (Takara, Guangzhou, China). Individual samples were measured in duplicate. A standard curve using serial dilutions of the pooled sample (cDNA from all samples), negative control without cDNA template, and a negative control without reverse transcriptase (RT) were included in every assay. Only standard curves with efficiency between 90% and 110% and a correlation coefficient above 0.99 were accepted. Data were normalized against the reference gene β-actin. Divergent primers annealing at the distal ends of circRNAs were used to determine the abundance of circRNAs, while the convergent primers were used to measure the mRNA content. To further confirm the junction sequence of circRNAs, PCR products of divergent primers were gel purified and handed in for Sanger sequencing at Sangon Biotech Co. Ltd. (Shanghai, China). The primers used and PCR annealing temperatures for each gene are summarized in Table S3.

### 4.10. Immunohistochemistry

Frozen ovaries (*n* = 6 from six different animals) were serial sectioned (7 µm). Serial sections from each of the six ovaries were selected at random and mounted on Superfrost plus glass slides (Menzel- Gläser, Braunschweig, Germany). To determine the presence of proteins (p53, PHLDA3, and cCASP3) in porcine ovaries, immunohistochemistry was performed, according to Meng et al., with some modifications [5,26]. For each antibody tested, all ovarian sections were stained in one run, in order to be able to compare the immunohistochemical staining among the different animals. Briefly, sections were air-dried for 30 min and fixed in 4% phosphate-buffered paraformaldehyde for 10 min. Slides were subsequently washed in water, microwaved in sub-boiling 0.1 M sodium citrate buffer (pH = 6) for 10 min for epitope antigen retrieval. Slides were cooled down to room temperature and rinsed with Tris-buffered saline (TBS) pH 7.4. Endogenous peroxidase activity was blocked with 3% (v/v) hydrogen peroxide in methanol. Aldehyde residues were blocked with 0.3% glycine in TBS for 30 min. After rinsing with TBS, sections were incubated with 5% (*wt/v*) normal goat serum in TBS for 60 min at room temperature. Following the removal of goat serum, sections were incubated overnight at 4 °C in a humidified chamber with the respective primary antibodies (p53, 1:200; PHLDA3, 1:200; cCASP3, 1:1000) diluted in TBS-BSAc (Aurion, Wageningen, The Netherlands). Sections were rinsed and incubated at room temperature with the corresponding secondary biotin-labeled antibody diluted 1:200 in TBS-BSAc. After rinsing with TBS, the avidin-biotin complex (ABC kit elite, Vector Laboratories, Burlingame, CA, USA) was diluted 1:750 (*v/v*) in TBS-BSAc. Bound antibodies were visualized using 3-3′ diaminobenzidine (Impact DAB kit, Vector Laboratories) diluted 1:400 (v/v). Sections were counterstained with Mayer’s hematoxylin. Control sections were incubated with isotype IgG (Vector Laboratories, Burlingame, CA, USA), instead of the respective primary antibodies, according to the manufacturer’s instructions. Background staining in these controls was negligible.

### 4.11. Statistical Analysis

To perform the statistical analysis, as described above, GraphPad Prism version 7.00 (Graphpad Software, San Diego, CA, USA) was used. Data were expressed as the mean ± standard error of the mean (SEM). Data were checked for normality, and when the normality was confirmed, the Student’s t-test was used for data analysis. If normality could not be assumed, data were log10 transformed. If data was still not normally distributed after log transformation, a Mann–Whitney non-parametric test was used. *p*-values < 0.05 were considered to be significantly different.

## 5. Conclusions

Taken together, our study provided an overview of the presence of circRNAs in granulosa cells from HA and AA follicles. The association between the expression of two differentially expressed candidate circRNAs (circ_CBFA2T2 and circ_KIF16B) and their respective targeted genes *GCLC* and *TP53* implicated a potential role for these circRNAs in granulosa cell apoptosis. The identification of RNA circularization may further expand our understanding of the molecular regulation atresia in antral follicles.

## Figures and Tables

**Table 1:** Characterization of DE-circRNAs with fold change more than 3 in porcine granulosa cells between healthy and atretic antral follicles.

Gene Name	FDR Value	Fold Change	Up/Down	Catalog	No of Exons	Length	Chromosome
circ_IL1R1	0.046	20.64	up	sense overlapping		50,710	chr3
circ_KIF16B	0.004	13.56	up	exonic	5	482	chr17
circ_UNKL	0.024	10.60	up	exonic	4	565	chr3
circ_PSPC1	0.04	8.33	up	exonic	4	680	chr11
circ_WDR7	0.049	7.99	up	exonic	2	859	chr1
circ_NCOR1	0.045	6.63	up	sense overlapping		64,858	chr12
circ_PTBP3	0.036	6.34	up	exonic	2	286	chr1
circ_MORC3	0.037	6.18	up	exonic	3	350	chr13
circ_GMPS	0.029	5.87	up	exonic	4	499	chr13
circ_MATR3	0.045	5.76	up	exonic	6	972	chr2
circ_LSM14A	0.045	3.72	up	sense overlapping		2856	chr6
circ_MCC	0.045	3.67	up	sense overlapping		67,640	chr2
circ_ZMYM4	0.036	21.30	down	exonic	5	670	chr6
circ_FAM49B	0.011	16.48	down	exonic	3	311	chr4
circ_ANGPT1	0.019	13.53	down	exonic	6	908	chr4
circ_MFSD14A	0.004	13.33	down	exonic	2	243	chr4
circ_ZC3H6	0.012	13.15	down	sense overlapping		4764	chr3
circ_SLC30A7	0.017	13.01	down	exonic	6	660	chr4
circ_CBFA2T2	0.007	12.63	down	exonic	4	768	chr17
circ_LOC100155760	0.012	12.02	down	antisense		17,825	chr1
circ_POLDIP3	0.017	11.92	down	sense overlapping		1227	chr5
circ_HCN1	0.012	11.78	down	sense overlapping		788	chr16
circ_SMAD2	0.006	11.57	down	sense overlapping		4357	chr1
circ_VPS50	0.045	11.03	down	sense overlapping		16,465	chr9
circ_CSDE1	0.005	10.67	down	exonic	2	208	chr4
circ_ANKHD1	0.006	10.34	down	exonic	6	936	chr2
circ_RB1	0.003	9.82	down	exonic	8	793	chr11
circ_GOLGA4	0.002	9.58	down	exonic	6	471	chr13
circ_STIP1	0.007	9.30	down	exonic	3	351	chr2
circ_NDUFB2	0.003	8.72	down	sense overlapping		7158	chr18
circ_EMC2	0.019	8.28	down	exonic	5	444	chr4
circ_ABHD17B	0.04	8.05	down	exonic	2	650	chr1
circ_SNX6	0.011	7.80	down	sense overlapping		328	chr7
circ_TMEM39A	0.021	7.80	down	exonic	2	307	chr13
circ_CSPP1	0.023	7.73	down	sense overlapping		23,646	chr4
circ_RNaseP_nuc	0.043	7.57	down	sense overlapping		131	chr7
circ_GCLC	0.04	7.39	down	exonic	5	603	chr7
circ_EED	0.041	7.34	down	exonic	4	367	chr9
circ_ PPP3CC	0.036	7.10	down	sense overlapping		665	chr14
circ_CPEB2	0.003	7.08	down	antisense		56,995	chr8
circ_SLC25A37	0.033	5.62	down	sense overlapping		59,863	chr14
circ_FAM188A	0.045	5.41	down	exonic	8	707	chr10
circ_ENSSSCG13774	0.042	4.99	down	sense overlapping		42,224	chr2
circ_N4BP2	0.04	4.91	down	sense overlapping		14,757	chr8
circ_CDH2	0.045	4.56	down	exonic	11	1323	chr6
circ_PPP3CB	0.04	4.14	down	exonic	8	1023	chr14
circ_ENSSSCG00025785	0.019	3.46	down	sense overlapping		33,804	chr13
circ_ELL2	0.046	3.42	down	exonic	3	473	chr2
circ_MELK	0.037	3.41	down	exonic	6	611	chr1
circ_POR	0.04	3.41	down	sense overlapping		367	GL894019-2
circ_ENSSSCG00021189	0.012	3.32	down	sense overlapping		81,337	chr11
circ_NUP155	0.007	3.16	down	exonic	2	272	chr16

## Supplementary Materials

Supplementary Materials can be found at https://www.mdpi.com/1422-0067/21/15/5217/s1. Figure S1. Representative immunostaining of cCASP3 (brown staining) as a marker of apoptosis in healthy (A) and atretic (B) antral follicles (*n* = 6 follicles from different animals). (A) Healthy antral follicles with no cCASP3 staining in the granulosa cells; (B) Atretic antral follicles with numerous granulosa cell-derived apoptotic bodies, adjacent to and within the antrum, that stain positively for the presence of cCASP3 (detail shown in insert). Granulosa cells are indicated by an asterisk, theca cells by an arrowhead. Scale bar represents 50 µm. Figure S2. Characteristic profiles of circRNAs and DE-circRNAs. (A) Length distribution for exonic circRNAs. (B) Distribution of circRNAs by chromosome. (C) The proportion of different types of DE-circRNAs. (D) The size distribution of DE-circRNAs. (E) Number of DE-circRNAs produced by each chromosome. DE-circRNAs, differentially expressed circRNAs. Figure S3. GO and KEGG analysis performed for the host genes of the DE-circRNAs. Two-D bar graph of biological process (A), molecular function (B), and cellular component (C) categories; bubble chart of potential signaling pathways (D). The length and color of each bar represent the number of genes and *p*-value, respectively. Similarly, the size and color of each bubble represent the number of genes in each pathway and *p*-value, respectively. DE-circRNAs, differentially expressed circRNAs. *n* = 3. Figure S4. CircRNAs-miRNAs interaction network. The V shape nods with red and green color represent circRNAs, and ellipse nodes (blue color) represent miRNAs. The red and green color denotes the up-regulation and down-regulation of circRNAs, respectively. The grey solid line represents the interaction between circRNAs and miRNAs, and the thickness of the line indicates the combined score. Figure S5. Representative GO terms of targeted genes of circ_CBFA2T2 (A) and circ_KIF16B (B). The size and color of each bubble represent the number of genes in each pathway and *p*-value, respectively. Table S1 All the significant GO terms; Table S2 KEGG pathway analysis of host genes of DE-circRNAs; Table S3 Sequences of the primers used for qRT-PCR.
