# Supplementary material for: Characteristics of Circular RNA Expression Profiles of Porcine Granulosa Cells in Healthy and Atretic Antral Follicles
Source: Int J Mol Sci. 2020 Jul 23;21(15):5217. doi: 10.3390/ijms21155217 (PMC7432752; doi:10.3390/ijms21155217)
Supplement: Supplementary file 1 [file ijms-21-05217-s001.zip › Supplemental Table 1.docx]

Supplemental 1 All the significant GO terms

| GO Term | P value | GENES |
| --- | --- | --- |
| regulation of homeostatic process | 0.001 | PPP3CB//GCLC//SLC30A7 |
| regulation of ion homeostasis | 0.006 | GCLC//SLC30A7 |
| vasculature development | 0.009 | ANGPT1//MIB1//PPP3CB |
| gene expression | 0.010 | SMAD2//NR5A2//RB1//ELL2//PTBP3//CPEB2//GCLC//PPP3CB |
| cellular homeostasis | 0.011 | GCLC//SLC30A7//PPP3CB |
| RNA metabolic process | 0.012 | SMAD2//NR5A2//RB1//ELL2//PTBP3//GCLC//PPP3CB |
| transcription, DNA-templated | 0.013 | NR5A2//RB1//ELL2//GCLC//PPP3CB//SMAD2 |
| nucleic acid-templated transcription | 0.013 | SMAD2//NR5A2//RB1//ELL2//GCLC//PPP3CB |
| RNA biosynthetic process | 0.014 | SMAD2//NR5A2//RB1//ELL2//GCLC//PPP3CB |
| cellular nitrogen compound biosynthetic process | 0.019 | SMAD2//NR5A2//RB1//ELL2//CPEB2//GCLC//PPP3CB |
| immune effector process | 0.019 | PPP3CB//TMEM39A//LOC100155760 |
| regulation of nitrogen compound metabolic process | 0.022 | NR5A2//RB1//GCLC//CPEB2//PPP3CB//PTBP3 |
| cellular macromolecule biosynthetic process | 0.022 | SMAD2//NR5A2//RB1//ELL2//CPEB2//GCLC//PPP3CB |
| regulation of gene expression | 0.022 | NR5A2//RB1//GCLC//CPEB2//PPP3CB//PTBP3 |
| nucleic acid metabolic process | 0.023 | SMAD2//NR5A2//RB1//ELL2//PTBP3//GCLC//PPP3CB |
| cardiovascular system development | 0.024 | ANGPT1//MIB1//PPP3CB |
| circulatory system development | 0.024 | ANGPT1//MIB1//PPP3CB |
| chemical homeostasis | 0.025 | SLC30A7//PPP3CB//GCLC |
| macromolecule biosynthetic process | 0.025 | SMAD2//NR5A2//RB1//ELL2//CPEB2//GCLC//PPP3CB |
| aromatic compound biosynthetic process | 0.026 | SMAD2//NR5A2//RB1//ELL2//GCLC//PPP3CB |
| heterocycle biosynthetic process | 0.027 | SMAD2//NR5A2//RB1//ELL2//GCLC//PPP3CB |
| organic cyclic compound biosynthetic process | 0.031 | SMAD2//NR5A2//RB1//ELL2//GCLC//PPP3CB |
| regulation of immune effector process | 0.031 | PPP3CB//TMEM39A |
| cellular nitrogen compound metabolic process | 0.035 | SMAD2//NR5A2//RB1//ELL2//PTBP3//CPEB2//GCLC//PPP3CB |
| defense response to virus | 0.037 | TMEM39A//LOC100155760 |
| regulation of RNA metabolic process | 0.041 | NR5A2//RB1//GCLC//PPP3CB//PTBP3 |
| response to stimulus | 0.041 | PPP3CB//TMEM39A//GCLC//SMAD2//MIB1//SLC30A7//CPEB2//ANKHD1//ANGPT1//LOC100155760 |
| anatomical structure formation involved in morphogenesis | 0.042 | ANGPT1//MIB1//PPP3CB |
| response to virus | 0.045 | TMEM39A//LOC100155760 |
| nucleobase-containing compound metabolic process | 0.046 | SMAD2//NR5A2//RB1//ELL2//PTBP3//GCLC//PPP3CB |
| heart development | 0.049 | MIB1//PPP3CB |
| response to endogenous stimulus | 0.050 | SMAD2//GCLC//CPEB2 |
