# Supplementary material for: Characteristics of Circular RNA Expression Profiles of Porcine Granulosa Cells in Healthy and Atretic Antral Follicles
Source: Int J Mol Sci. 2020 Jul 23;21(15):5217. doi: 10.3390/ijms21155217 (PMC7432752; doi:10.3390/ijms21155217)
Supplement: Supplementary file 1 [file ijms-21-05217-s001.zip › Supplemental Table 2.docx]

Supplemental table 2 KEGG pathway analysis of host genes of DE-circRNAs

| Pathway | P value | GENES |
| --- | --- | --- |
| HTLV-I infection | 0.000 | IL1R1//PPP3CB//RB1//SMAD2 |
| Pancreatic cancer | 0.004 | RB1//SMAD2 |
| Apoptosis | 0.008 | IL1R1//PPP3CB// SMAD2 |
| Oocyte meiosis | 0.012 | CPEB2//PPP3CB |
| Cell cycle | 0.013 | RB1//SMAD2 |
| Osteoclast differentiation | 0.017 | IL1R1//PPP3CB |
| Alzheimer's disease | 0.029 | NDUFB2//PPP3CB |
| Maturity onset diabetes of the young | 0.035 | NR5A2 |
| Dorso-ventral axis formation | 0.041 | CPEB2 |
