# Supplementary material for: Characteristics of Circular RNA Expression Profiles of Porcine Granulosa Cells in Healthy and Atretic Antral Follicles
Source: Int J Mol Sci. 2020 Jul 23;21(15):5217. doi: 10.3390/ijms21155217 (PMC7432752; doi:10.3390/ijms21155217)
Supplement: Supplementary file 1 [file ijms-21-05217-s001.zip › Supplemental Table 3.docx]

Table 1 Sequences of the primers used for qRT-PCR.

| **Circle Gene symbol** | **Primers** | | **Anealing temp (°C)** | **Length of product** |
| --- | --- | --- | --- | --- |
| circ_RB1 | Forward | TAATTGCACCGTGAATCCAA | 60.00 | 219 |
|  | Reverse | TCACCAGATCATCTTCCATTTG |  |  |
| circ_CBFA2T2 | Forward | CTCCGCCTCTTCAGCACTAC | 60.00 | 190 |
|  | Reverse | GCAGGACCATTGCTGAATCT |  |  |
| circ_WDR7 | Forward | CTACCAGGCCACCTAGACCA | 60.00 | 201 |
|  | Reverse | CAACGCTTCCACATCAAAGA |  |  |
| circ_SLC30A7 | Forward | TGTAATTGCCTCTGCCATCA | 60.00 | 170 |
|  | Reverse | TAACAGATGCTGCCAATCCA |  |  |
| circ_ANGPT1 | Forward | GGAAACCGAGCCTATTCACA | 60.00 | 199 |
|  | Reverse | AGCTTTCTGGTCTGCTCTGC |  |  |
| circ_GMPS | Forward | TGATGGATTCAAGGTTGTGG | 58.00 | 190 |
|  | Reverse | TCCAAGGGGAAGATTTCAGA |  |  |
| circ_KIF16B | Forward | CATGACCGACCTCTCCAAGT | 60.00 | 203 |
|  | Reverse | CTGCCAACATACGTCTGACC |  |  |
| circ_ANKHD1 | Forward | GATGCTGGTGCAGATCAAGA | 60.00 | 204 |
|  | Reverse | TGCTTCTAGTCGTGCCTGTG |  |  |
| circ_UNKL | Forward | CGGTTCCCATACAGGTCCAC | 60.00 | 205 |
|  | Reverse | GGTGCCCGTTTTGTAGTAGC |  |  |
| circ_ZMYM4 | Forward | AAGGGGAAACCGAACAGG | 57.00 | 68 |
|  | Reverse | TTCAGGGCATCCGTTTGT |  |  |
| circ_IL1R1 | Forward | ATTTGGGGCTGGGGATAG | 60.00 | 153 |
|  | Reverse | TTTCATCTTCACGCTCCTCA |  |  |
| ACTB* | Forward | TGGAACGGTGAAGGTGACAG | 60.00 | 175 |
|  | Reverse | CTTTTGGGAAGGCAGGGACT |  |  |
| KIF16B | Forward | CTTCAGGACTTGCGTTGTGC | 60.00 | 125 |
|  | Reverse | CGAGACACCATCCGCTCATA |  |  |
| CBFA2T2 | Forward | GTTGGAGAGCTTGGCGGC | 60.00 | 145 |
|  | Reverse | CTTTTGTCAGCACCAAGCTGGA |  |  |
| SLC30A7 | Forward | GAGTCGAGAGAGCCTTAGCC | 60.00 | 219 |
|  | Reverse | TCAGTTCATGACCGTGGCAA |  |  |
| ANGPT1 | Forward | TCATGCTAACAGGAGGCTGG | 60.00 | 213 |
|  | Reverse | CTCTGTGGCTTTTACGATCACT |  |  |
| HIP1 | Forward | CCCCTAGGATCCCACCTGAA | 60.00 | 204 |
|  | Reverse | CCAGGAGACCCTGCTAGACT |  |  |
| TP53INP1 | Forward | TAAGGGCAGGGCATCAGTT | 60.00 | 174 |
|  | Reverse | GCTCAAGAAAACAAGCTGCCA |  |  |
| Frzb | Forward | AGCAGTGAACGCTGCAAATG | 60.00 | 207 |
|  | Reverse | GCCAGAGCTGGTGTAAAGGT |  |  |
| RPS6KA2 | Forward | GCCCTCGTCCTAAAAGCCAA | 60.00 | 167 |
|  | Reverse | TTCCAGTCGATGGTGGCAAA |  |  |
| Hdac4 | Forward | GAGCCAGTGGAGACCCCT | 60.00 | 185 |
|  | Reverse | AAAGTCCATCTGGGTGGCTC |  |  |
| TP53 | Forward | CTATAGCGATGGTCTGGCCC | 60.00 | 104 |
|  | Reverse | CCACAACGCTGTGTCGAAAA |  |  |
| IKBKB | Forward | GACGCTGGACGACCTAGAAG | 60.00 | 191 |
|  | Reverse | CCTTCTGCTTGCAAACCACC |  |  |
| SGPL1 | Forward | CGCTATAGCATGTGGGCATCG | 60.00 | 147 |
|  | Reverse | TGCAGAGCGCTGTACAAACT |  |  |
| PTGIS | Forward | CAGCAGCATCAAACAGTTCGT | 60.00 | 220 |
|  | Reverse | CACAGAAAGTTGGGCGACTG |  |  |
| LTBR | Forward | ACCACCTGCAGAAATCCACC | 60.00 | 187 |
|  | Reverse | TTGATTCCTCTCCCTCTGGGT |  |  |
| SGPP1 | Forward | ACCAAGGACATCATCCGCTG | 60.00 | 167 |
|  | Reverse | GCAATGACATCCTGCCAACG |  |  |
| GCLM | Forward | ACCAGTGGGCACAGGTAAAA | 60.00 | 200 |
|  | Reverse | CCACTCATGTGCCTCGATGT |  |  |
| DHCR24 | Forward | CGATGCACACCGTCAGAAAA | 60.00 | 204 |
|  | Reverse | TTCCACGAAGTGGTTCTCGG |  |  |
| IL33 | Forward | ACCAGATCACAAGAAGCCTG | 60.00 | 89 |
|  | Reverse | GCCGGCTGAGCTATTCATCT |  |  |
| SLC25A4 | Forward | TGATACTGCCAAGGGGATGC | 60.00 | 133 |
|  | Reverse | CATCATCCTACGGCGGACAG |  |  |
| GCLC | Forward | GATCCTCCAGTTCCTGCACA | 60.00 | 87 |
|  | Reverse | GAGAGAGAACCAACCTCGTCG |  |  |
| PUMA | Forward | GCAGAAAACCGAGCGAGAGT | 60.00 | 110 |
|  | Reverse | CTCCCTGGGGCCATGAATC |  |  |
| BIM | Forward | CAGAGCGGCAAGCTTCCAT | 60.00 | 139 |
|  | Reverse | AAAGAAAACAGCATTACCCTCCTTG |  |  |
| PHLDA3 | Forward | GTGGTGTCAGCCCCCTG | 60.00 | 184 |
|  | Reverse | CCATCCCGAGAGTCTGCATC |  |  |

Gene denoted with an asterisk was used as reference gene for normalization.

Abbreviations: *RB1*, RB transcriptional corepressor 1; *CBFA2T2,* CBFA2/RUNX1 partner transcriptional co-repressor 2; *WDR7*, WD repeat domain 7; *SLC30A7*, Solute carrier family 30 member 7；*ANGPT1*，Angiopoietin 1；*GMPS*, guanine monophosphate synthase; *KIF16B*, kinesin family member 16B; *ANKHD1*, ankyrin repeat and KH domain containing 1; *UNKL*, unk like zinc finger; *ZMYM4*, zinc finger MYM-type containing 4; *IL1R1*, interleukin 1 receptor, type I; *ACTB*, actin beta; *HIP1,* huntingtin interacting protein 1; *TP53INP1,* tumor protein p53 inducible nuclear protein 1; *TGFB2,* transforming growth factor beta 2; *Frzb,* frizzled related protein; *RPS6KA2,* ribosomal protein S6 kinase A2; *TP53,* tumor protein p53; *IKBKB,* inhibitor of kappa light polypeptide gene enhancer in B-cells kinase beta; *SGPL1,* sphingosine-1-phosphate lyase 1; *PTGIS,* prostaglandin I2 synthase; *LTBR,* lymphotoxin beta receptor; *SGPP1*, Sphingosine-1-phosphate phosphatase 1; *IL33*, interleukin 33; *GCLM*, glutamate-cysteine ligase; *DHCR24*, 24-dehydrocholesterol reductase; *SLC25A4*, Solute carrier family 25 member 4; *GCLC*, glutamate-cysteine ligase catalytic subunit; *PUMA*, BCL2 binding component 3; *BIM*, phorbol-12-myristate-13-acetate-induced protein 1; *PHLDA3*, pleckstrin homology like domain family A member 3.

.
