# Supplementary figures and images for: Characteristics of Circular RNA Expression Profiles of Porcine Granulosa Cells in Healthy and Atretic Antral Follicles
Source: Int J Mol Sci. 2020 Jul 23;21(15):5217. doi: 10.3390/ijms21155217 (PMC7432752; doi:10.3390/ijms21155217)

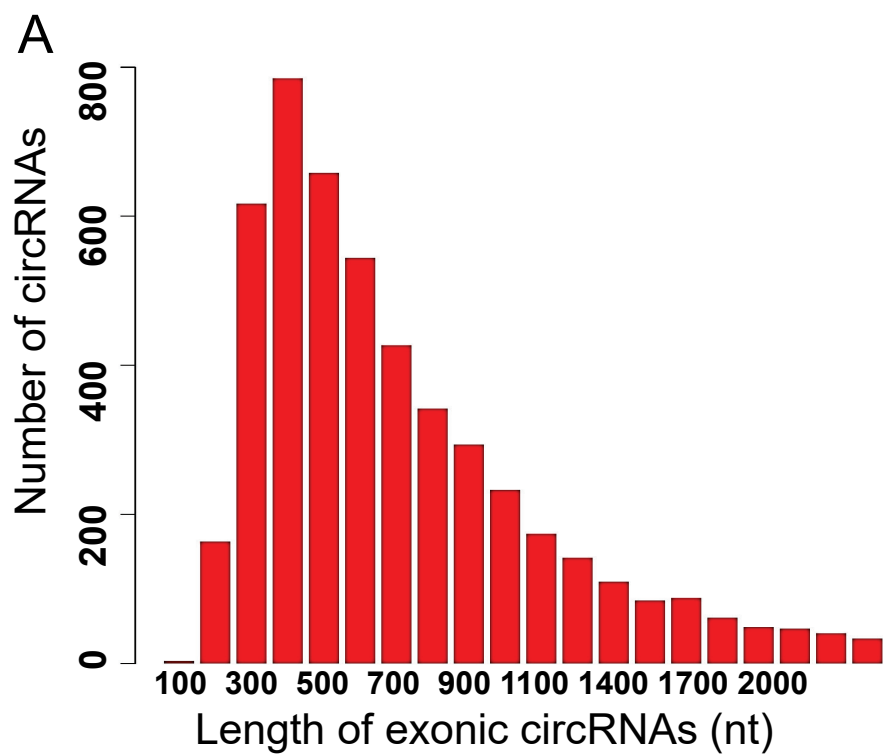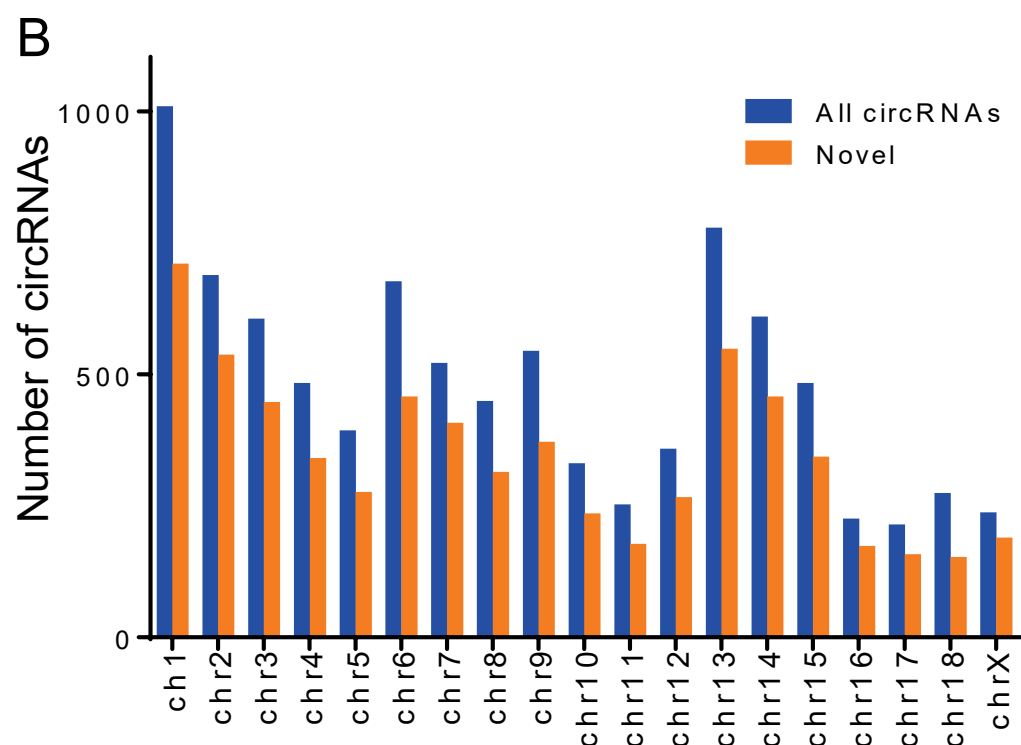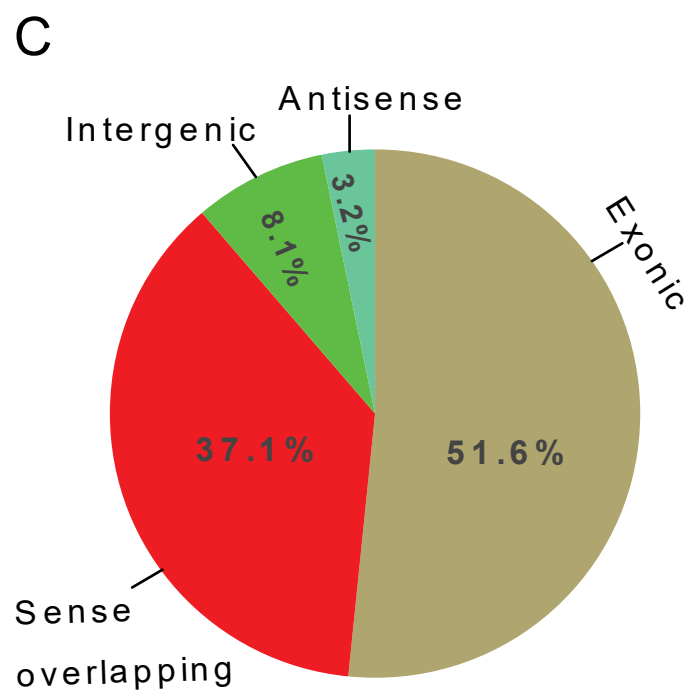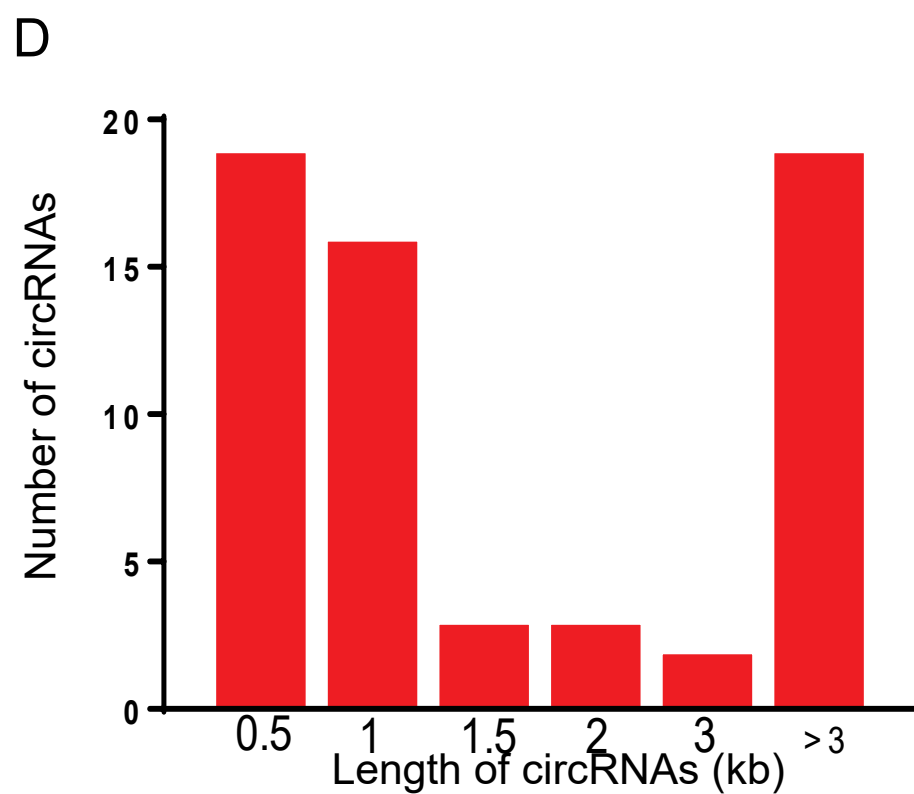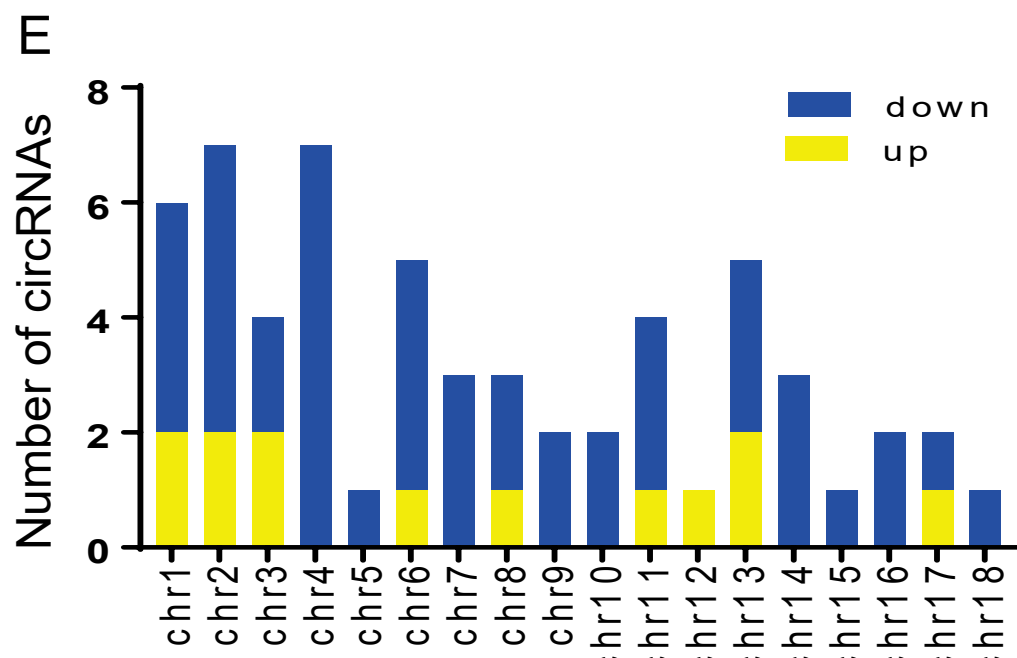

Supplement: Supplementary file 1 [file ijms-21-05217-s001.zip › Supplemental Figure 2.pdf]

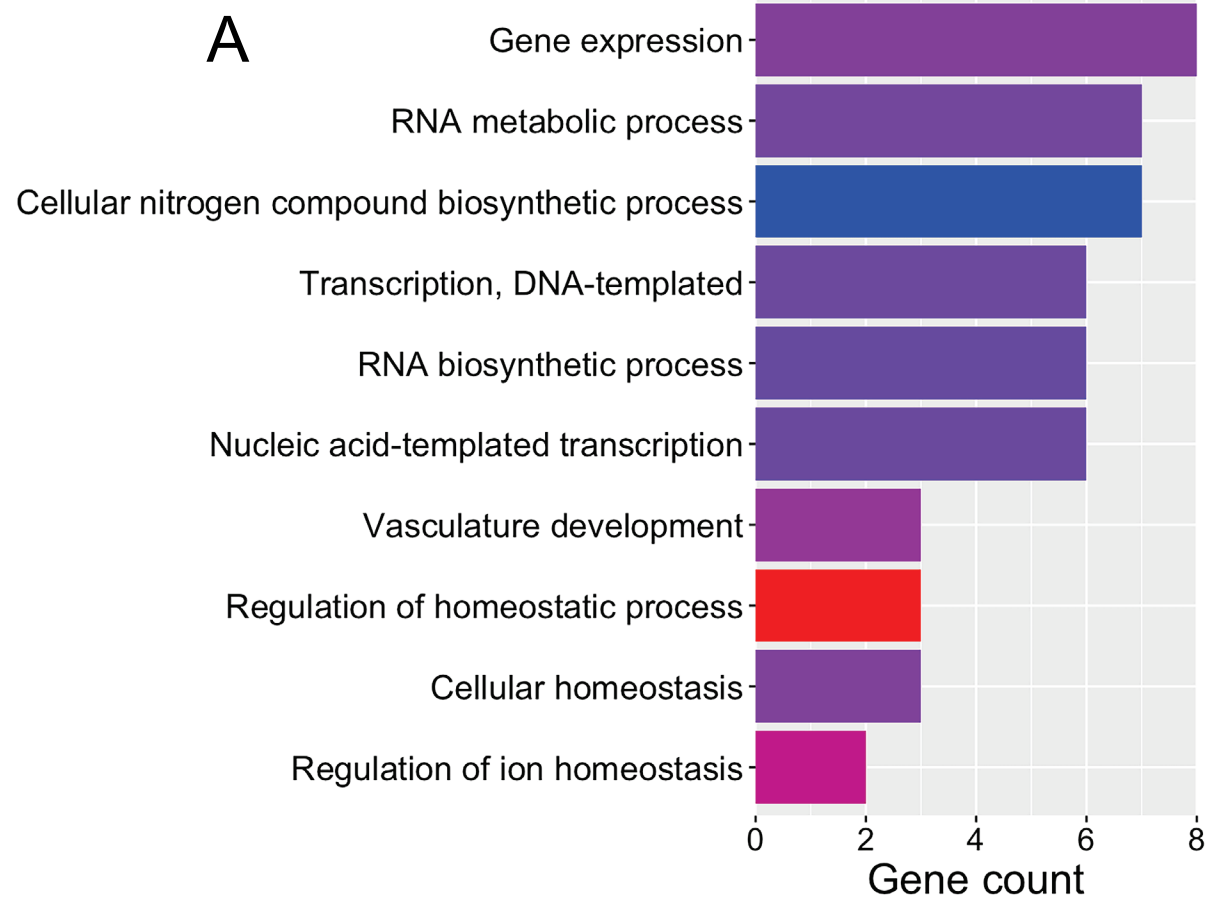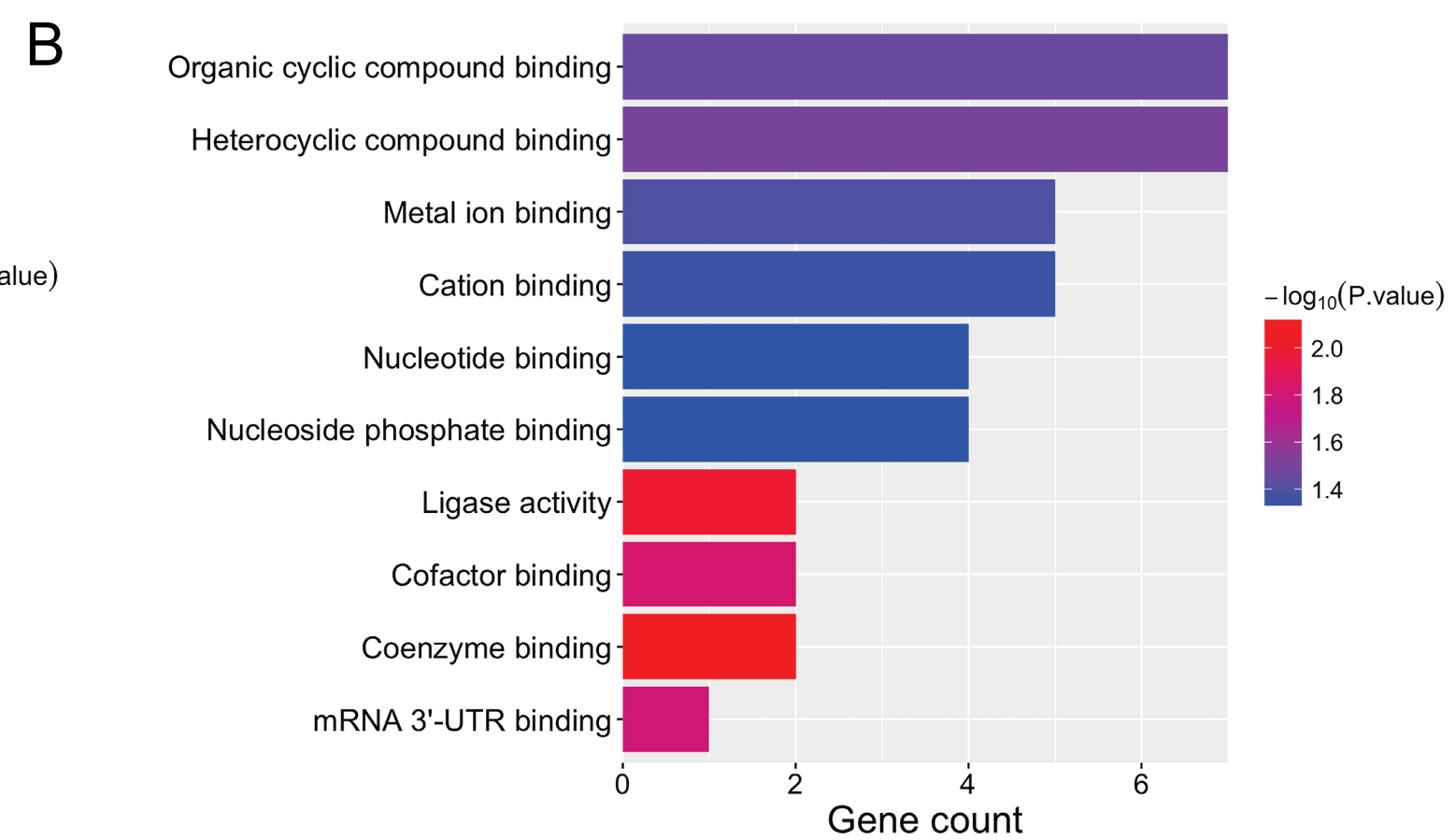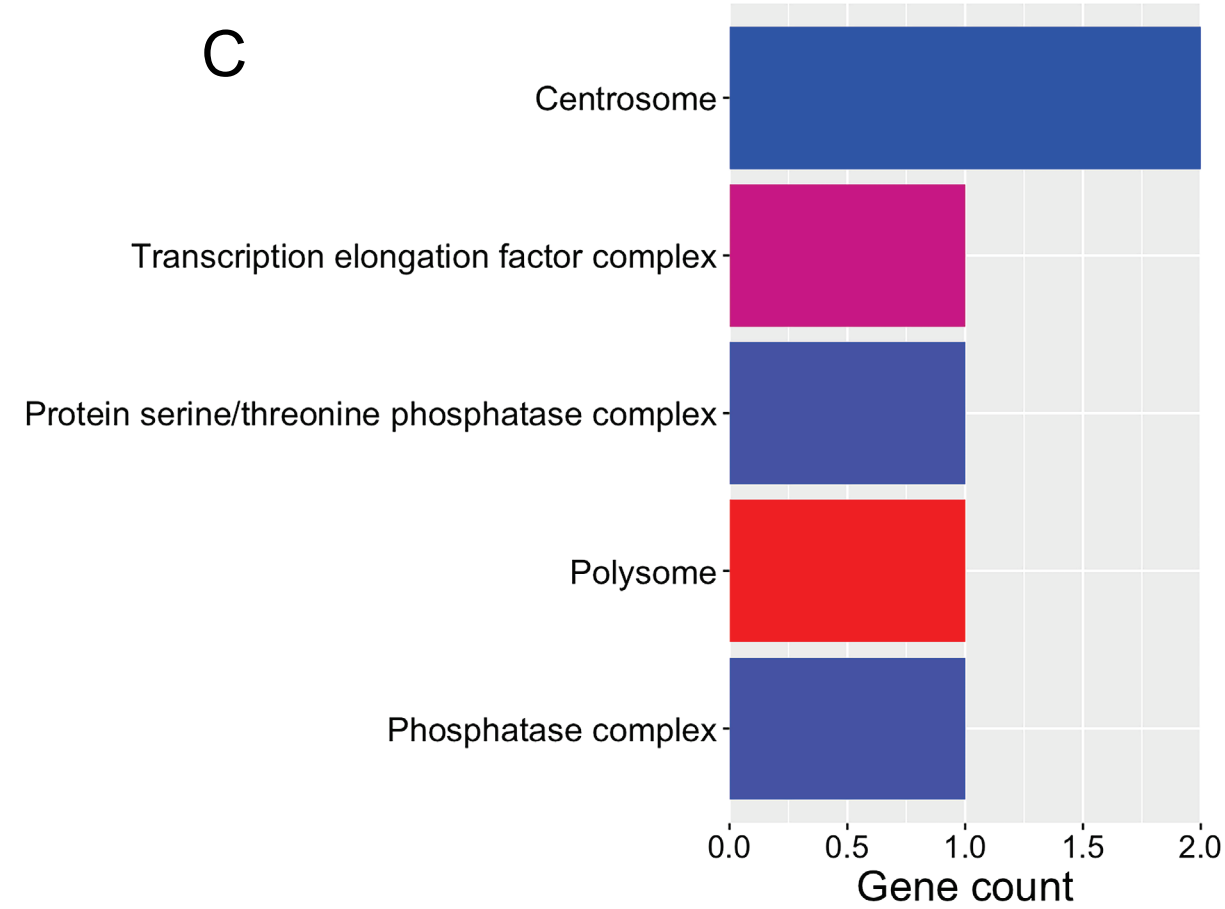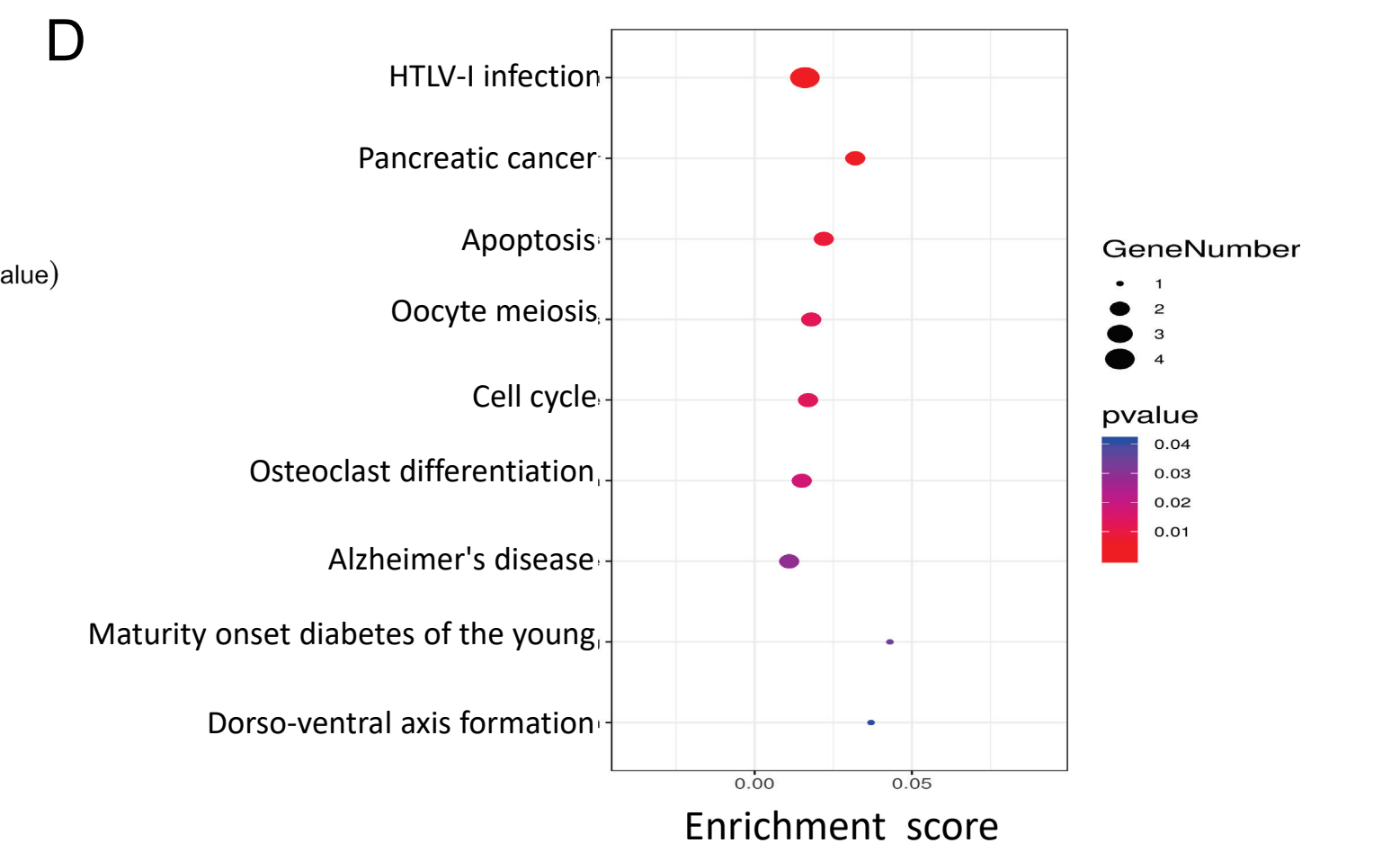

Supplement: Supplementary file 1 [file ijms-21-05217-s001.zip › Supplemental Figure 3.pdf]

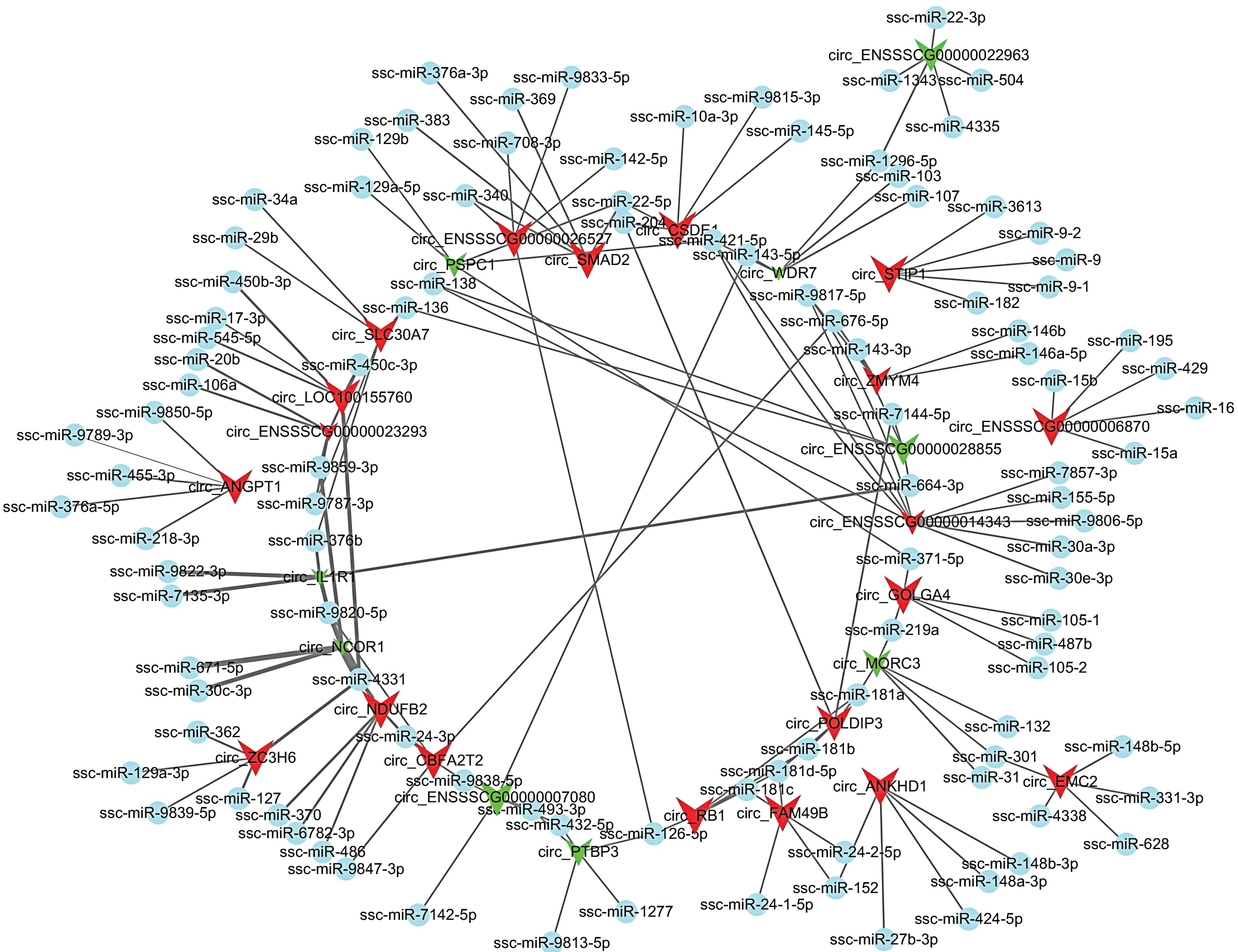

Supplement: Supplementary file 1 [file ijms-21-05217-s001.zip › Supplemental Figure 4.pdf]

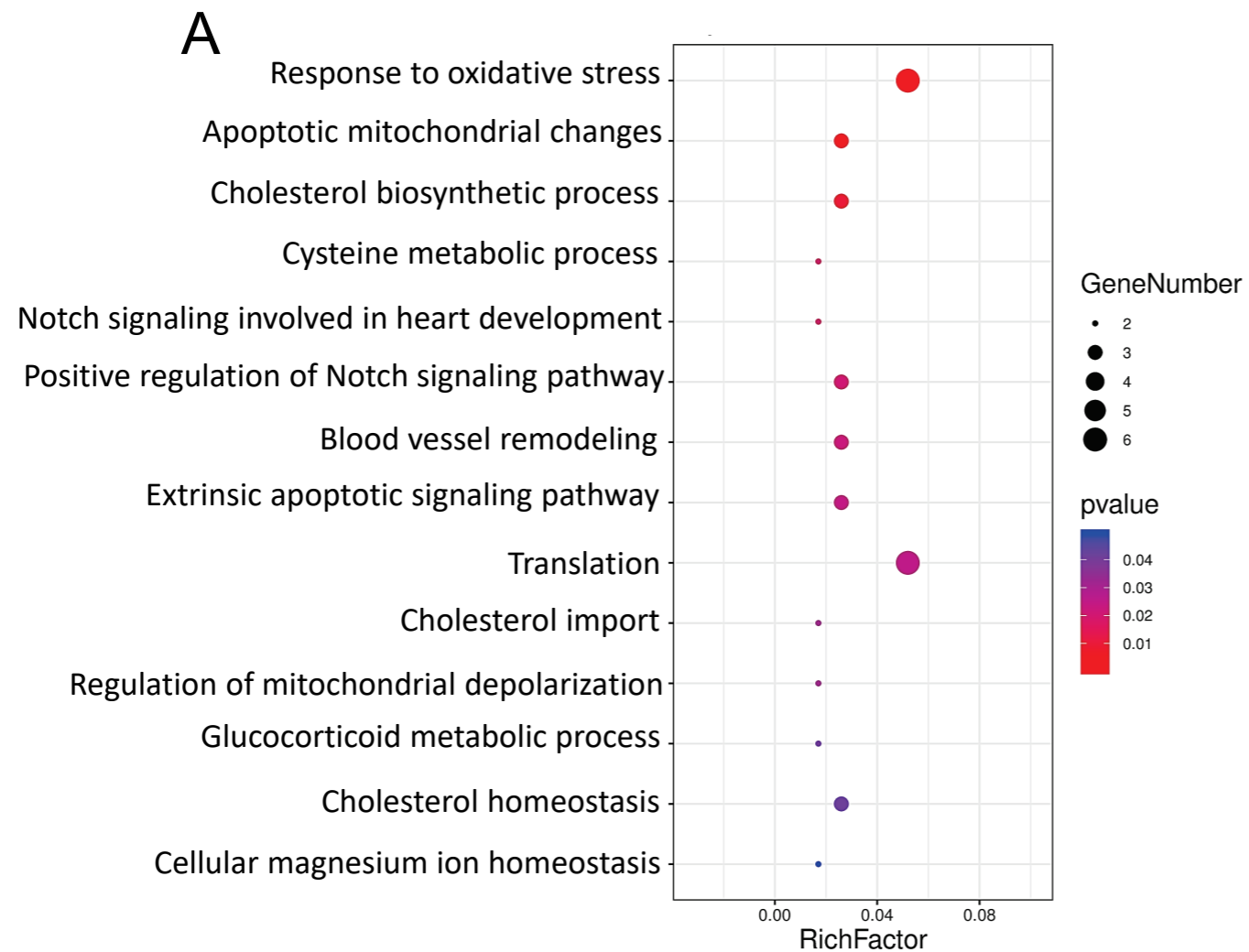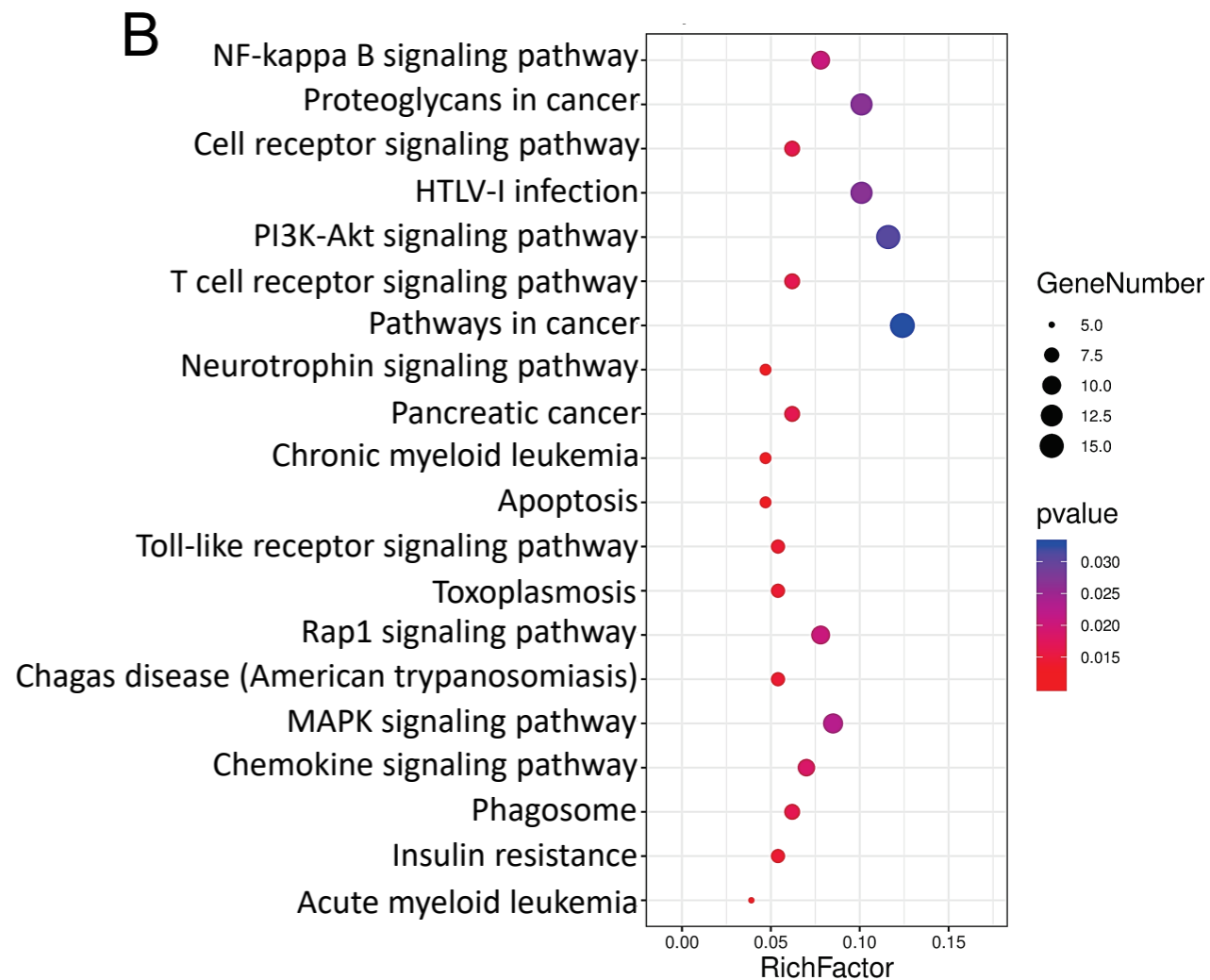

Supplement: Supplementary file 1 [file ijms-21-05217-s001.zip › Supplemental Figure 5.pdf]

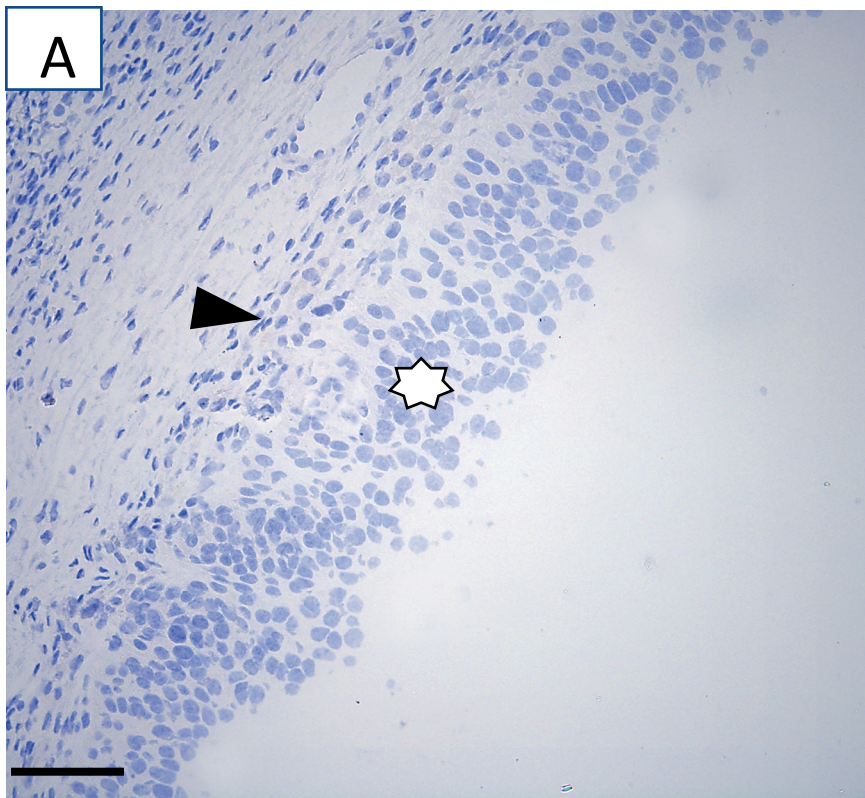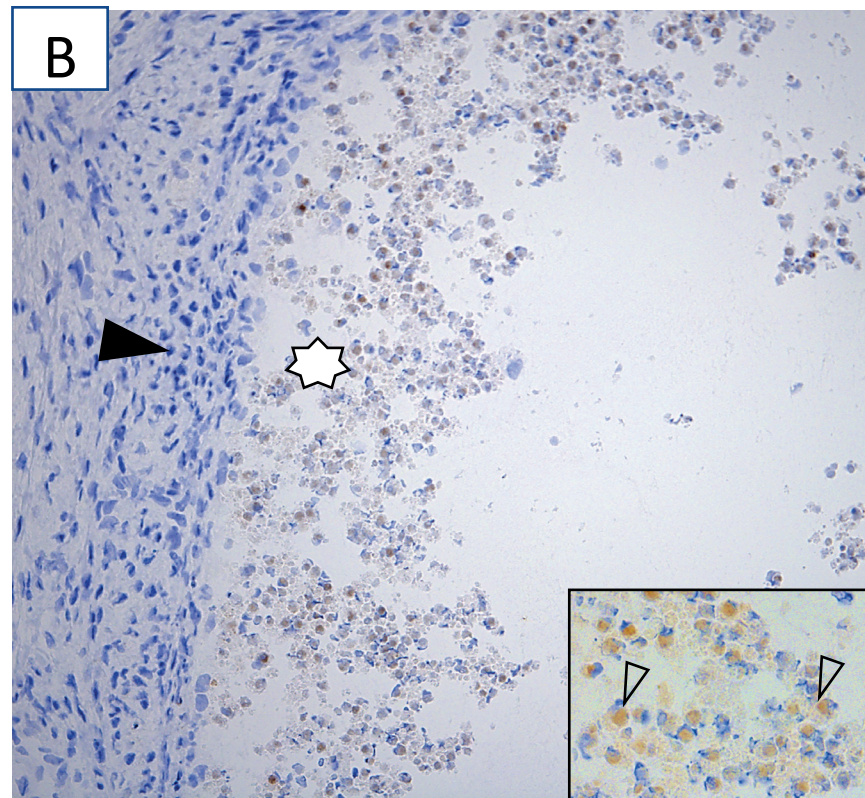

Supplement: Supplementary file 1 [file ijms-21-05217-s001.zip › Supplemental Figure 1.pdf]
